# Did trade unions protect employees’ mental health during the COVID-19 pandemic? A mixed effects model using UK data from Understanding Society

**DOI:** 10.1136/bmjph-2024-001756

**Published:** 2025-05-21

**Authors:** Theocharis Kromydas, Evangelia Demou, Alastair H Leyland, Srinivasa Vittal Katikireddi, Jacques Wels

**Affiliations:** 1 MRC/CSO Social and Public Health Sciences Unit, University of Glasgow, Glasgow, UK; 2 Health & Society Research Unit, Université libre de Bruxelles (ULB), Brussels, Belgium; 3 Unit for LifeLong Health and Ageing (LHA), University College London, London, UK

**Keywords:** Epidemiology, Public Health, Occupational Medicine, Sociodemographic Factors

## Abstract

**Introduction:**

Few studies have addressed the relationship between trade unions and workers’ mental health during the COVID-19 pandemic.

**Methods:**

We analysed panel data from Understanding Society collected before and during the COVID-19 pandemic (49 915 observations; 5988 respondents) to assess the relationship between union presence within the workplace and union membership and a binary measure of common mental disorders (CMD), the 12-Item General Health Questionnaire (>4, probable psychological distress). A mixed-effect log-linear model assessed effect heterogeneity across time and industries, with average marginal effects (AME) indicating effect differences between groups.

**Results:**

Of our sample, 49.1% worked in a unionised workplace, with 53.8% of them being union members. Approximately 25% of the entire workforce was trade union members. Psychological distress prevalence was higher during the pandemic (25.4%) compared with prepandemic (18.4%). Union presence ((AME_pre-pandemic_: 1.0, 95% CI−0.66 to 2.70) (AME_-pandemic_: −0.2, 95% CI−1.91 to 1.58)) and union membership ((AME_pre-pandemic_: 1.6, 95% CI −0.69 to 3.93) (AME_pandemic_: −0.1, 95% CI −2.29 to 2.00)) were both associated with modest protection against CMD risk. Although, industry heterogeneity exists.

**Conclusions:**

Trade union presence may have a protective effect on workers’ mental health in periods of crisis, such as during a pandemic. Within unionised workplaces, trade union membership further mitigated the negative effects of the pandemic on mental health. Collective negotiation may be protective in periods of uncertainty, benefiting all workers.

**WHAT IS ALREADY KNOWN ON THIS TOPIC**

- There is a limited number of studies addressing the relationship between trade unions and workers’ mental health during the COVID-19 pandemic. Previous studies show that trade union presence within the workplace is associated with positive health outcomes, while union membership is associated with mixed findings.
- We do not know the extent to which trade unions might have protected people’s mental health during the COVID-19 pandemic characterised by a global increased burden on people’s mental health and instability.

**WHAT THIS STUDY ADDS**

- We use UK longitudinal data to address the change in workers’ mental health between pre- and pandemic times depending on union presence and union membership.
- Both trade union presence and membership are associated with lower probabilities of mental health decline during the pandemic.

**HOW THIS STUDY MIGHT AFFECT RESEARCH, PRACTICE OR POLICY**

- Trade unions may have a protective effect on workers’ mental health in periods of crisis, but industry heterogeneity exists.

## Introduction

Mental health is a major policy concern with recent events, including the COVID-19 pandemic, having long-lasting effects on populations.^1^ In such a context, employment is often seen as a determinant of mental health^2 3^ with precarious forms of employment having detrimental effects.^4^ However, while the role of employment characteristics in explaining population mental health is well documented,^5^ studies on the association between collective negotiation and the role of trade unions and workers’ health are very few. An emerging amount of evidence has pointed out the relationship between workers’ health and the role of trade unions. Studies have shown that workplace collective negotiation is associated with better workers’ health outcomes and that the lack of such negotiation is often associated with greater vulnerability within the workplace.^6^ The absence of a trade union or staff association within the workplace is associated with both poorer workers’ physical and mental health.^7^ However, the nature of this relationship is complex, and the few studies on the topic are contradictory with some demonstrating a negative relationship between trade union membership and mental health. One of the reasons for conflicting results is the level of collective negotiation that is taken into consideration in explaining population health. While the general tendency would be to focus on union membership—that is, whether a worker is an actual member of a trade union—other studies have emphasised that the role of trade unions within workplaces goes beyond membership behaviours. In that sense, unions would also contribute to explain the health of those who are not unionised.

Three types of approach exist. A first set of studies—that is the most common among the literature—pays attention to the relationship between union membership and health using cross-sectional, cross-sectorial or macrolevel data. For instance, it has been recently demonstrated using comparative cross-sectional data that health inequalities are high when unions only represent part of the workforce but low when a high proportion of the workforce is unionised.^8^ Similarly, high country trade union density (ie, calculated based on union membership rates) is associated with lower depressive symptoms among the workforce.^9^ The same type of analyses was also made looking at differences across sectors of activity based on union densities.^10 11^

A second type of studies takes a collective approach by focusing on the negotiating process within health and safety committees that exist in most OECD countries. Those committees are set up to negotiate within the workplace (but also at sector level) working conditions and safety matters and involve trade unions (when the company has trade union representatives) or workers’ representatives. For instance, using Korean cross-sectional data, it was shown that health and safety committees reduce work accidents but seem to be more effective in unionised workplaces.^12^ By contrast, Bryson has shown for the UK that union representation within health and safety committees is linked with lower health and safety risks compared with non-unionised workplaces.^13^ More recently, a study has linked company-based social dialogue quality and workers’ health as perceived by trade union representatives during the pandemic.^14^

A third type of studies has very recently focused on the individual relationship between union membership—and, to a lesser extent, the presence of a trade union within the workplace—and health mainly using individual longitudinal data. These studies have demonstrated contradictory results that show either a positive^7 15^ or a negative relationship,^16^ mainly due to the nature of the variables used to capture trade union presence or membership and the control variables included in the models. A few other studies have focused on the benefits of using a longitudinal approach to assess the association between union membership and wages^17^ or job satisfaction^18^ or the relationship between cumulative unionisation over the life course and health among older adults.^19^ These are important factors as the benefits of unions on wages, pensions, job security, workplace discrimination or healthcare coverage reflect on health.^20^ Such a perspective is still rare when looking at health and particularly mental health. This is the approach adopted in the study.

Yet, most of these approaches have an individualistic view on unions, assuming that any potential benefits gained apply only to members. As many studies distinguish union members and non-union members, the indirect role of union presence in company-based collective negotiations or health and safety committees in protecting those unionised as well as those not unionised has been ignored.^21^ One of the reasons for conflicting results is the level of collective negotiation that is taken into consideration in explaining population health.^21^ While the general tendency would be to consider union membership—ie, whether a worker is an actual member of a trade union—as the exposure,^8 16^ other studies have emphasised that the role of trade unions within workplaces goes beyond membership behaviours.^21^ In that sense, unions would also contribute to explain the health of those who are not unionised.^8^ Studies have underlined that union presence—that measures whether union representatives are involved in collective bargaining and health and safety committees at a workplace level—is a more relevant distinction because it includes the potential health benefits that affect those in a unionised workplace but are not members of a trade union.^7 22^

The distinction between trade union membership and trade union presence is of interest when looking at the UK for two major reasons. First, the UK currently has a highly company-based collective bargaining system with most negotiation taking place at workplace or company level and very few at sector level.^7^ In other words, trade unions act as collective institutions aiming to protect the interest of their members.^23^ However, their presence and actions affect everyone in the workplace (both members and non-members). Second, the relative decline in union membership rates—that is particularly due to structural changes, the rise in non-standard employment and the decline of typically unionised industries^24^ but also to a shift in personal values^25 26^—have contributed to consider unions as not policy relevant anymore. In the UK, despite having increased slightly over the past few years, mainly driven by a surge in female members, membership rates were around 23.7 percent in 2020.^27^ By contrast, compared to union membership levels,^28^ the percentage of employees in the UK covered by a union or workplace council has slightly dropped from 50.2 to 48.2 percent between 1996 and 2018. In other words, the number of workers working in a unionised workplace is more than twice the number of affiliated members,^7^ and one out of two workplaces still has at least one representative trade union.^7^

This study specifically focuses on addressing the role of both trade union presence and trade union membership in explaining mental health within the workforce in the specific context of the COVID-19 pandemic. The pandemic was a period of high economic disruption, restrictions and changes in employment settings and practices occurred, including an abrupt increase in home working^29^,^31^ and the use of temporary unemployment schemes (furlough)^32^ that had an important effect on population health. While the relationships between change in employment status and work pattern and workers’ mental health have been documented throughout this period,^31 32^ less is known about the way workplace characteristics—and, particularly, trade union presence—may have affected mental health. Trade unions played a crucial role during such a period, advocating for worker safety, protection of jobs and fair compensation for those affected by the economic fallout of the pandemic.^33 34^ COVID-19 has been seen as an occupational disease, and unions have worked to advocate and protect the workforce in key sectors of activity.^35 36^ While social dialogue between social partners and the state has been slow in delivering policies or action during the pandemic in the UK,^37^ the role of trade unions was pivotal at company-level where collective negotiation, participation in Safety and Health Committees^13^ and greater job stability^38^ might have had a protective role on working conditions. A report by the Trade Union Congress highlighted how unions remained active during the pandemic and increased the number of health and safety representatives in an effort to reduce the impacts of the pandemic on workers.^39^

Few studies have examined the relationship between workers’ mental health and trade unions during the pandemic and prepandemic.^7 15 16 21^ Social dialogue played a key role in protecting the workforce against COVID-19 infections,^14^ and unions were also efficient in moderating COVID-19 cases, as evidenced in a study based on US data.^40^ A questionnaire survey in Philadelphia (USA) collected in summer 2020 has highlighted that perceived support from trade unions was associated with lower depressive symptoms and anxiety.^41^ Similarly, a Belgian questionnaire study has shown that trade union representatives reported better mental health across their company workforce when social dialogue was of a rather good quality.^14^ Another questionnaire survey in Latvia has shown that trade union presence was associated with higher odds of depression when implementing home working policies; however, the survey suffers from gender imbalance as most participants were female (85%).^42^ The current study focused on workers’ mental health changes observed between a period of a health crisis such as the COVID-19 pandemic where population and workers’ mental health deteriorated and a period of time before the pandemic started.^3043^,^46^ The primary research objective of the study is to address whether workers in unionised workplaces experienced the same changes in mental health during the pandemic compared with workers in non-unionised workplaces. Additionally, we investigate whether any changes were observed by sector of activity and whether the same patterns are revealed when union membership is used as an exposure instead of trade union presence. By addressing these, the study aims to evidence the nature of the relationship between trade unions and workers’ mental health, during major crises, such as the recent pandemic compared with prepandemic period.

## Materials and methods

### Understanding Society (USoc)

We used data from Understanding Society (USoc), a large-scale, longitudinal social survey in the UK that collects data on individuals and households across various topics, including health, education, employment and social attitudes, to inform research and policymaking. Understanding Society has conducted 14 waves of data collection, starting in 2009.

We specifically used waves 9, 10 and 11 (baseline sample covering the period from January 2017 to March 2020) and all nine COVID-19 sweeps collected throughout 2020 and 2021: sweep 1 (April 2020), sweep 2 (May 2020), sweep 3 (June 2020), sweep 4 (July 2020), sweep 5 (September 2020), sweep 6 (November 2020), sweep 7 (January 2021), sweep 8 (March 2021) and sweep 9 (September 2021). The analytical sample was restricted to respondents who participated in at least one of prepandemic waves (waves 9, 10, 11), were employed at that time (excluding self-employed workers—but not those who combined employment and self-employment) and also participated in, at least, one of the nine COVID-19 sweeps (see online supplemental file S1).

USoc uses probability-based sampling for all components of the study; however, not all population groups are selected with the same probability. To correct for this bias, a series of design weights exist, and USoc strongly recommends the user to include these where applicable. USoc also uses methods to maximise response rates and minimise non-response bias to ensure the participating sample represents well the initially selected sample. Such methods include boosted samples for population groups that are notoriously underrepresented in survey responses such as immigrant and ethnic minority groups. Responses across time were collected using different modes such as face-to-face, telephone-based interview but also online (COVID-19 sweeps).

### Patient and public involvement

None.

### Outcome variables

The 12-Item General Health Questionnaire (GHQ-12) caseness (ie, psychological distress) was our outcome of interest. It is derived from the GHQ-12 based on the answers to 12 questions that assess the severity of mental problems over the past few weeks.^47^ More specifically, GHQ-12 includes answers in terms of frequencies (ie, never, sometimes, often, very often) to 12 questions on being able to concentrate, sleep loss, feeling under strain, feeling not being able to overcome difficulties, feeling unhappy and depressed, losing confidence, feeling as a worthless person, feeling like not playing a useful role in life, not feeling capable to make decisions about things, not being able to enjoy normal day-to-day activities, not being able to face up to problems and not feeling reasonably happy. Valid answers are converted to a single scale by recoding values and then summing them, giving a scale running from 0 (the least distressed) to 12 (the most distressed). The GHQ-12 variable is a validated screening tool for identifying common mental disorders (ie, probable anxiety and/or depression), and it was used on a binary basis (GHQ-12 caseness or not) based on a cut-off score of 4.^48^

### Exposure variables

We used two exposure variables and replicated the analyses for each. *Union presence* was defined by participants’ yes-no response to the following question: “is there a trade union, or a similar body such as a staff association, recognised by your management for negotiating pay or conditions for the people doing your sort of job in your workplace?’. We also used the *union membership* variable that applies only to respondents in workplaces where there is a union or staff association and distinguished between members and non-members. Data on union presence and union membership for the employed workforce were only collected in prepandemic waves 2, 4, 6, 8 and 10. We carried forward union presence and union membership variables’ values for waves 9 and 11 using available data in waves 8 and 10, respectively. Then, we carried all data forward to all COVID-19 sweeps. This data generation technique was based on the relative stability of occupation and industry across time^44 49^ and by testing changes in union presence and membership over time in the prepandemic period. The latter showed very high overall stability across waves, which supports our assumption that carrying data forward will be unlikely to bias our results (see online supplemental file S1, table S1.1 and S1.2).

### Covariates

The model controls for a set of socioeconomic and demographic confounders. *Gender* (female (ref) and male); *Age* as a continuous variable and a quadratic function of age; *Ethnicity* (non-white, white (ref)); *Country* of residence (Scotland, Wales, Northern Ireland and England (ref)); Industry group (Major Standard Industrial Classification group (ref: hotel and accommodation); highest level of *education* achieved (A-level, General Certificate of Secondary Education (GCSE), other, none or university degree (ref));^28^ *Company size* (1 to 34 (ref), 25 to 199, more than 200); self-reported *financial situation* was used as a dummy variable distinguishing those living comfortably, doing all right combined (coded as 0, ref.) and those finding it very difficult, difficult to cope or just about getting by; *Heath status* (reporting a long-standing illness or impairment: yes (ref.), no); and dummy variables for waves (ref: wave 9). Variables were measured at each time point apart from self-reported financial situation, health status, company size, industry and level of education where data were carried forward from the closest available wave. The health status reflects respondents’ report of a long-standing (more than 12 months) physical or mental impairment, illness or disability to reflect baseline health. Moreover, data for the self-reported financial situation were carried one COVID sweep backwards when non-available in the current sweep or previous ones. We included dummy variables for waves of participation to account for secular effects across time, and we further include an *Industry* variable including 15 levels for the Standard Industry Classification as done previously ^44^ as an effect modifier.

### Statistical analysis

We used a mixed-effects analysis with a log-linear link function (for binary outcomes) accounting for repeated observations over time on the individual level and adjusting for autocorrelation of observations within individual values using the cluster sandwich estimator, which allows for intragroup correlation in standard errors. We estimated unadjusted and adjusted models using both odds ratios (reported in the online supplemental file S2) and predicted probabilities (predicted marginal means and average marginal effects). The unadjusted model (model 1) regresses our binary outcome variable on our exposures by using two-way interactions between trade union presence/membership and a binary variable that shows prepandemic (waves 9, 10 and 11) and pandemic periods (all COVID-19 sweeps).

The regression equation for model 1 is as follows:


$${GHQ-12case}_{iw}=a_{0}+{\gamma X}_{iw}+\theta Z_{iw}+\mu_{iw}+\pi_{i}+\varepsilon_{iw}$$

where i represents individuals, w represents waves/Sweeps, α_ο_ is the intercept, γ represents the vector of our exposure groups as explained above and θ represents a vector of all our additional covariates. Fixed effects at the individual and wave levels are represented by μ_iw_, π_i_ denotes the subject-specific random effects, while ε_iw_ is the error term that varies randomly across waves/sweeps. When ORs are estimated, interaction effects are calculated in a multiplicative manner (Model 1 logit(P{GHQ-12_case_=1})^time period*trade union presence/membership^ and Model 2 logit(P{GHQ-12_case_=1}^time period*industry* trade union presence/membership^), whereas marginal means represent additive effects (Model 1 (P{GHQ-12_case_=1})_time period+ trade union presence/membership_ and Model 2 (P{GHQ-12_case_=1}_time period+industry+ trade union presence/membership_).

The adjusted model 2 includes the same two-way interaction but controls for the full set of covariates. Finally, we included an industry classification term creating a three-way interaction, which was our effect modifier in model 3. Analyses were replicated using USoc-provided sampling weights with no major difference across estimates. Additionally, we calculated stabilised Inverse Probability Weights (IPW) for our outcome missingness separately for each analytical sample used and wave/sweep based on basic demographics (age, sex, UK country of residence, ethnicity and trade union presence or trade union membership). This results to each individual assigned a weight that is the inverse of their probability of being a complete case. The weights used for our final estimates were the product of the USoc-provided sampling weights and the stabilised IPW we calculated for outcome missingness.

To better interpret the estimates flowing from the logistic regression models, we converted the ORs to probabilities (additive instead of multiplicative effects) using the Stata *margins* command focusing on the fixed part of the mixed-effects regression equation. We have estimated both the marginal means (MM)—that is, the predicted value of the dependent variable when one independent variable changes, while others remain unchanged—and average marginal effects (AME), that is, the average change in the dependent variable for a unit change in the independent variable of interest, averaged across all observations in the dataset.

### Sensitivity analyses

We conducted six sensitivity analyses. First, we excluded trade union members to check the specific relationship between union presence and GHQ-12 caseness for the non-unionised workforce. Second, we excluded publicly owned workplaces (where the proportion of unionised workplaces in our data is larger compared with private-owned workplaces). Both models were replicated on the adjusted models including a two-way and a three-way interaction. Third, we replicated the adjusted model with a two-way interaction using union presence as the exposure of interest but controlling for union membership. Fourth, we replicated the adjusted model with a two-way interaction using union membership at the exposure of interest but controlling for union presence. Fifth, we ran a linear model using a variable that measures mental health on a broader scale (GHQ-36) as our outcome variable. We calculated the MMs for the last two models. We identified no differences in the direction of the effect compared with our binary logistic model. Finally, as our financial hardship variable may be on the causal pathway between unions and psychological distress because unions are associated with wages, we have estimated a model for trade union presence excluding the variable on financial situation to measure its influence on our exposure estimates. We found that estimates remain very similar (online supplemental file table S4.8).

## Results

The study included 49 915 observations among 5988 participants (total sample) and 27 971 observations among 3341 participants after restricting the sample to those reporting union presence and running the analysis using trade union membership as our exposure (online supplemental file S1).

Table 1 shows that, among the respondents who were in employment at data collection time, 50.8 per cent reported having a trade union within their workplace (weighted data) before the start of the pandemic against 48.5 per cent during the pandemic. In other words, union presence applied to about half of the sample. When looking at the population working in a unionised workplace, we observe that trade union membership is 57.4 per cent in prepandemic waves and 52.5 per cent during the pandemic. More than half of the workforce in unionised workplaces is a trade union or staff association member.

**Table 1:** Trade union presence and membership in pre-pandemic and pandemic waves (unweighted)

		Pre-pandemic	Pandemic	Total
Full sample	No trade union presence	6636 (46.9%)	16 958 (47.4%)	23 594 (47.3%)
	Trade union presence*	7506 (53.1%)	18 815 (52.6%)	26 321 (52.7%)
	Total	14 142 (100.0%)	35,773 (100.0%)	49,915 (100.0%)
Restricted only to workplaces where trade union is present†	No trade union membership	3106 (41.6 %)	9164 (44.7%)	12 270 (43.9%)
	Trade union membership	4361 (58.4%)	11 340 (55.3%)	15 701 (56.1%)
	Total	7,467 (100.0%)	20,504 (100.0%)	27,971 (100.0%)

* There is a small difference between Trade Union presence counts and Total counts for trade union members. This is due to some respondents having reported working in place where trade union is present but missed to report their trade union membership status.

† trade union presence is collected among the full sample whilst trade union membership is only asked to respondents who reported trade union presence.

Further descriptive statistics on sample composition are shown in online supplemental file S3) (online supplemental tables S3.1, S3.2). For our analytical sample including both unionised and non-unionised workplaces, psychological distress was 17.7 per cent before the pandemic and 23.8 per cent during the pandemic (respectively 18.4 and 25.4 per cent when data are weighted). When data are restricted to workplaces where there is a trade union present, psychological distress is 18.6 and 24.4 per cent (19.4 and 25.3 per cent after weighting), pre and during the pandemic, respectively (online supplemental table S3.1).

We also observe a difference in industry representation depending on whether the sample is restricted to workplaces where a trade union is present. For instance, while the composition of workers in the public administration and defence industry is 10.1 and 7.2 per cent in the full sample in prepandemic and pandemic waves, these reach 17.0 and 11.2 when the sample is restricted to union presence. The same pattern can be observed in other industries, which are heavily represented by the public sector. For instance, sample composition for Education is 9.3 and 8.3 per cent higher in the restricted sample. Similarly, for Human Health and Social Work Activities, the equivalent compositional percentages were 5 and 4.6 (online supplemental table S3.2). This indicates that union presence in these industries is particularly salient.

In table 2, the AMEs refer to the difference in the prevalence of GHQ-12 caseness between respondents in unionised and non-unionised workplaces. They were estimated separately for the pre- and pandemic periods for both samples used. Negative AMEs denote higher prevalence for respondents in non-unionised workplaces (2a) and trade union non-members (2b). In the adjusted model 1, MMs show that the average GHQ-12 caseness prevalence in unionised workplaces is slightly higher pre-pandemic (12.1, 95% CI 10.45 to 13.83) compared with non-unionised workplaces (11.1; 95% CI 9.42 to 12.83), but this trend reverses in the pandemic period with respectively 14.8 (95% CI 13.51 to 16.14) and 15.00 (95% CI 13.51 to 16.14). This is reflected by the positive AME sign prepandemic (1.0, 95% CI −0.66 to 2.69) and the negative sign during the pandemic period (−0.2, 95% CI −1.91 to 1.58).

**Table 2:** Adjusted models (model 1) marginal means (MM) and average marginal effects (AVE) for trade union presence and trade union membership as exposures

		Trade union presence(full sample)						
Model 1	Time period	Trade union presence	MM	95% CI		AME	95% CI	
				Lower	Higher		Lower	Higher
	Prepandemic	No	0.111	0.076	0.439			
	Prepandemic	Yes	0.121	0.048	0.276	0.010	−0.007	0.027
	Pandemic	No	0.150	0.135	0.283			
	Pandemic	Yes	0.148	0.064	0.233	−0.002	−0.019	0.016

			Trade union membership(sample restricted to trade union presence)					
	Time period	Trade union membership	MM	95% CI		AME	95% CI	
				Lower	Higher		Lower	Higher
	Prepandemic	No	0.119	0.096	0.142			
	Prepandemic	Yes	0.135	0.111	0.158	0.016	−0.007	0.039
	Pandemic	No	0.155	0.138	0.172			
	Pandemic	Yes	0.154	0.137	0.170	−0.001	−0.023	0.020

Note: All models are adjusted for gender (ref.: female), age, age square, ethnicity (ref.: white), industrial group (ref.: hotel and accommodation), UK country of residence (ref.: England), education (ref.: university degree), financial situation (ref.: doing all right), baseline (pre-pandemic) health condition (ref.: longstanding illness or impairment) and size of workplace (ref.:1 to 34 employees). Secular effects across time are accounted for by the inclusion of dummy variables for each pre-pandemic Wave and COVID-19 Sweep

We replicated model 1 using union membership as an exposure variable within a sample that only includes respondents who worked in workplaces where a trade union staff association is present (table 2). An almost identical pattern was observed as for union presence but with even wider CIs. The prepandemic AME was 1.6 (95% CI −0.69 to 3.93), and the pandemic AME was −0.1 (95% CI −2.29 to 2.00).

**Figure 1:**
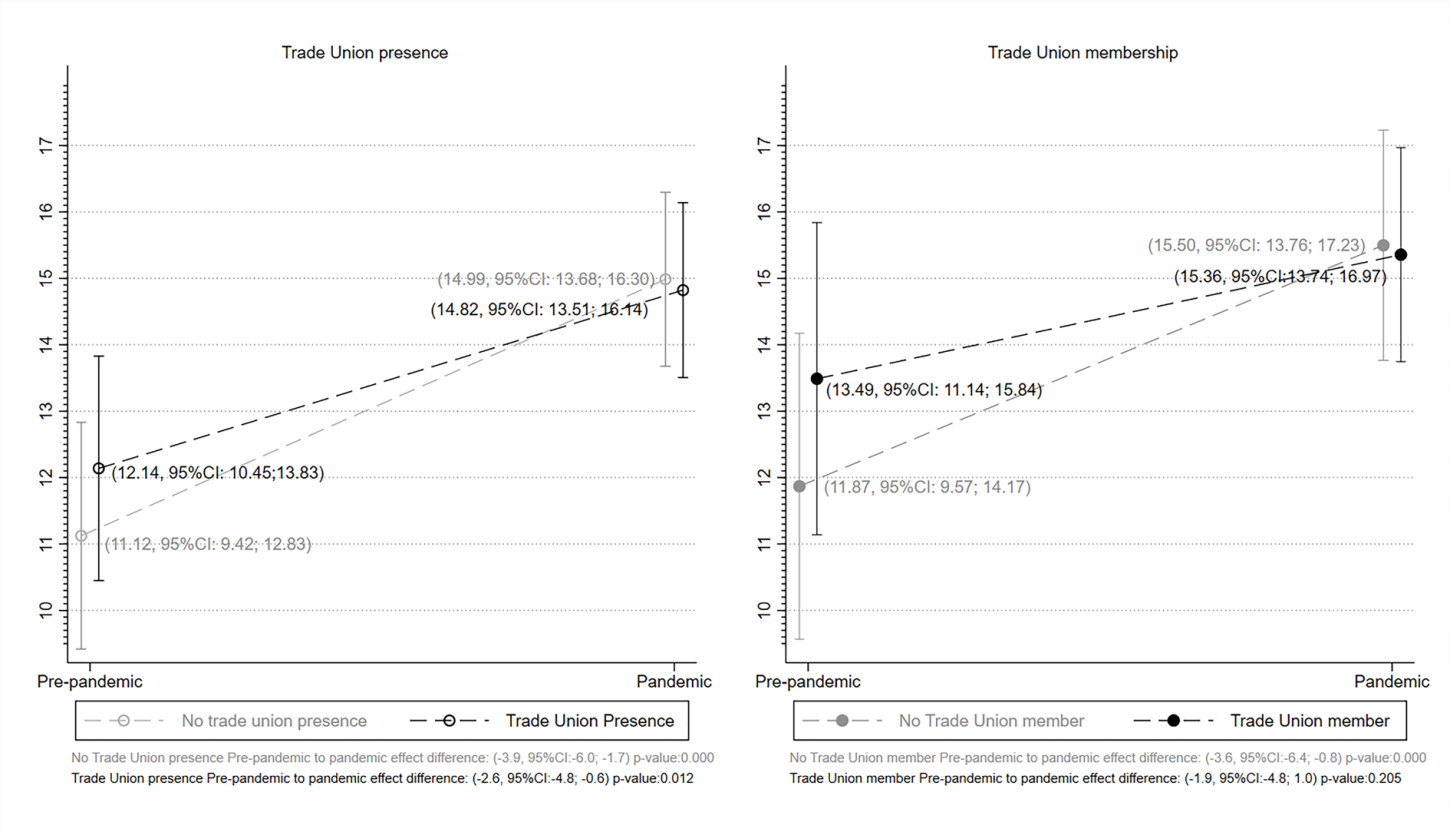
Marginal means of the 12-Item General Health Questionnaire (GHQ-12) caseness in prepandemic and pandemic periods by union presence and union membership.

These estimates are summarised in figure 1 which shows the marginal means for trade union presence (within the full working population) and trade union membership (within the population working in a unionised workplace) before the outbreak of the COVID-19 pandemic and during the pandemic. What can be observed is that the probability of psychological distress increased during the pandemic for all workers irrespective of whether they worked in a unionised workplace or were a member of a trade union. However, the increase for those working in a unionised workplace has been less sharp: the probability of psychological distress for those in workplaces without union presence was 11.1 per cent (95% CI 9.42 to 12.83) prior to the pandemic against 15.0 per cent (95% CI 13.68 to 16.30) during the pandemic, with non-overlapping CIs. By contrast, for the unionised workplaces, the equivalent difference shows confidence intervals that overlap. The probability of GHQ-12 caseness was 12.1 per cent (95% CI 10.45 to 13.83) prior to the pandemic and 14.8 per cent (95% CI 13.51 to 16.14) during the pandemic. This implies that union presence in workplaces has an apparent cushioning effect for the mental health of workers compared with union absence. When examining union membership as the exposure, differences across predictive margins are similar, but CIs become wider, principally because the sample was restricted to those in unionised workplaces. Our estimates show a difference between members and non-members before the start of the pandemic with a probability of 11.9 per cent (95% CI 9.57 to 14.17) for those who are not trade union members and 13.5 per cent (95% CI 11.14 to 15.84) for those who are. This difference seems to have reversed during the pandemic with respectively 15.5 per cent (95% CI 13.76 to 17.23) and 15.4 per cent (95% CI 13.74 to 16.97), indicating a potential cushioning effect of union membership, although CIs are wide and overlap.

What is observed above is an average result that does not consider the potential differential trends that could be observed across industries (see table 2 (bis) in online supplemental file S2). Once industry classification is added as a term of the interaction effect (model 2), we observe effect magnitude and direction variations of trade union presence (and membership) by industry, although with very broad CIs that do not allow us to draw meaningful conclusions in most groups due to sample limitations. In only just three (Accommodation and Food services (AME, 10.2%; 95% CI: −19.6; −0.1], Real Estate Activities [AME:21.5% CI: 3.5; 39.5] and Education [AME:5.1% CI: 1.2; 8.9]) CIs do not cross zero and also this stands only for the pre-pandemic period (table 2). Thus, we are unable to draw statistically robust conclusions regarding the comparison of the AMEs between the prepandemic and pandemic periods. When we use trade union membership as an exposure, CIs are too wide and cross zero in all industries.

Results from the sensitivity analyses are shown in online supplemental file S4. Excluding union members from the analyses when using trade union presence as the outcome of interest in online supplemental S4.1 and S4.2 or including union membership as a control variable in online supplemental S4.5 does not drastically affect the ORs. Similarly, excluding public workplaces from the analyses (online supplemental S4.3 and S4.4) does not affect the results that were observed in the original models. Online supplemental S4.10 plots the same marginal means as in figure 1 but controlling for union membership when union presence is the exposure of interest and for union presence when union membership is the exposure. MMs for workers with no trade union presence is 11.4 (95% CI 9.61 to 13.17) in prepandemic times compared with 15.4 (95% CI 13.96 to 16.85) during the pandemic, which denotes, as in the main model, a significant increase. By contrast, trade union presence shows MMs of 11.8 (95% CI 10.05 to 13.47) and 14.5 (95% CI 13.13 to 15.92) in both periods, showing a lower slope and a non-significant difference. When examining trade union membership within the full population, we also notice that non-union members experienced a slightly sharper increase in psychological distress compared with union members. Finally, sensitivity analyses also showed that using a linear modelling for GHQ-36 as an outcome instead of GHQ-12 caseness. We do not observe major changes in the direction of the estimates.

## Discussion

Two main findings flow from this study. First, we demonstrate that, while the whole workforce’s mental health declined after the start of the COVID-19 pandemic, the increase in psychological distress was proportionally higher among workers not employed in a workplace where there is a trade union or staff association. The same trend is observed for union membership (after restricting the sample to workers working in a unionised workplace only), but results are not robust enough to draw inferences. Second, heterogeneity exists across industries, and there is no consistent pattern on the effect of trade unions on mental health across time in any.

Few studies have explored the longitudinal relationship between trade unions and mental health, with most focusing solely on trade union membership and overlooking the significant role played by the presence of a trade union within the workplace. This article’s primary contribution lies in demonstrating similar associations for both variables, but with stronger effects observed for trade union presence. These findings suggest that unions may play a crucial role in promoting the health of workers, including those who are not unionised. This is particularly true in the context of crisis addressed in this study. While the relationship between changes in employment settings and workers’ health has been largely documented throughout the pandemic,^3150^,^52^ quantitative research published on the possible role of trade unions in providing cushioning to the deterioration of workers’ mental health during the COVID-19 pandemic is few, and the number of qualitative studies on this matter remains low, mostly theoretical and addressing the mechanisms of protection without estimating their prevalence for the entire workforce.^53^

This study has limitations, which can open the way for further research on collective negotiation and workers’ health.

First, Understanding Society did not collect information on trade union presence and membership and industry classification during the pandemic, and information had to be carried forward from prepandemic waves. Similarly, this stands for some of the covariates used. Industry or even company changes were not likely to occur to a large extent during the pandemic; however, they were not impossible, and this study overlooks these potential changes due to data limitations. It is possible that trade union membership could have actually changed; however, during a pandemic and especially during lockdowns, such changes are expected to be very limited due to harsh movement restrictions imposed by the government as well as the protective measures implemented (eg, furlough). Therefore, it is very likely that changes in union membership and presence were much more limited compared with prepandemic. Particularly for trade union presence, we expect no real changes during the pandemic compared with the prepandemic period. Trade union presence is at the workplace level, and therefore changes, if any at all, would likely happen at a much slower rate compared with changes in trade union membership which is at the individual level. This supports our strategy to carry data forward from the pre- to the pandemic period. To illustrate such stability, online supplemental file S.5 exhibits the percentage of trade union presence and membership across Understanding Society available waves as well as stability rates (ie, the percentage of change from one wave to another) showing relatively constant rates of both union presence and membership and relatively little individual change.

Second, the study did not account for changes in employment status throughout the pandemic. While respondents who moved to furlough (ie, the UK job retention scheme) are included in the sample, we did not account for those who became unemployed or were made redundant during the pandemic. This study specifically focuses on the differences in mental health across populations remaining in employment during the pandemic and therefore does not account for the potential protective effects of trade unions against redundancy or unemployment. Understanding how unions might secure employment trajectories and therefore reduce the mental health burden of job loss was not the focus of this study but would deserve further investigation.

A third limitation is that this study did not include information on home-working due to lack of data availability over the selected waves. While trade unions were reluctant to implement home-working policies before the pandemic^54^ and home-working propensities were lower within the unionised workforce,^55^ the specific effect of trade unions for those working from home is still to be investigated.

A fourth limitation is about the definition of trade union presence. No other variable on trade unions is available within the dataset, and we had to rely on self-reported information, and, since the definition of workplace union presence is self-reported, misclassification might happen. Furthermore, union presence does not guarantee de facto the presence of health and safety committees and translates into different workplaces’ practices.^56^

Finally, an important limitation is that we compared groups that are unlikely to be exchangeable with many potential residual confounding not measured in this study, as well as possible reverse causation.^57^ Trade union membership and, to a lesser extent, presence are not assigned randomly across individuals, and further studies should address this issue.

Nevertheless, this study demonstrates that beyond individual unionisation behaviours, collective negotiation at the workplace could have an alleviative mental health effect on the whole workforce and not only union members. It takes place in a current climate of restrictions in the power of collective bargaining in countries where trade unions are present. Furthermore, despite a slight increase in union membership rates in the UK over the past 2 years, the long-term world trends show a massive decrease in union membership.^58^ The COVID-19 pandemic has illustrated the importance of work and employment in shaping population health and well-being. This might encourage policy makers to promote collective bargaining and democracy at work to protect the workforce and improve population health by promoting individuals’ union membership, on the one hand, and facilitating collective negotiation on the other hand on the industrial/occupational level, especially during periods of crises.

## Supplementary material

10.1136/bmjph-2024-001756 online supplemental file 1

## Data Availability

Data are available upon reasonable request.
