# Supplementary material for: Did trade unions protect employees’ mental health during the COVID-19 pandemic? A mixed effects model using UK data from Understanding Society
Source: BMJ Public Health. 2025 May 21;3(1):e001756. doi: 10.1136/bmjph-2024-001756 (PMC12104941; doi:10.1136/bmjph-2024-001756)
Supplement: online supplemental file 1 [file bmjph-3-1-s001.docx]

**Supplementary files**

[Supplementary file 1. Flowchart of study participants 2](#_Toc188371066)

[Union presence flowchart 2](#_Toc188371067)

[Union membership flowchart 3](#_Toc188371068)

[Testing the stability assumption (Trade Union presence and Trade union membership variables) 4](#_Toc188371069)

[Table S1:Transitions between trade union presence values (Yes :Unionised workplace, No: Non-unionised workplace) 4](#_Toc188371070)

[Table S2:Transitions between trade union membership values (Yes :member, No: Non-member) 4](#_Toc188371071)

[Supplementary file S2 – Main estimates 5](#_Toc188371072)

[Union presence – unadjusted model 5](#_Toc188371073)

[Union presence – adjusted model (2-way interaction) 6](#_Toc188371074)

[Union presence – adjusted model (3-way interaction) 9](#_Toc188371075)

[Union membership – unadjusted model 14](#_Toc188371076)

[Union membership – adjusted model (2-ways interaction) 15](#_Toc188371077)

[Union membership – adjusted model (3-ways interaction) 18](#_Toc188371078)

[Table 2 (bis): Adjusted Models (Model 2, by industry) Marginal Means (MM) and Average Marginal Effects (AVE) for trade Union Presence and trade union membership as exposures 23](#_Toc188371079)

[Supplementary file S3. Sample characteristics for trade union presence and membership 27](#_Toc188371080)

[Table S3.1. Sample characteristics for union presence in the pre and pandemic USoc Waves* 27](#_Toc188371081)

[Table S3.2. Observations for Industry (SIC-2007) for union presence in pre-pandemic and pandemic Waves (Unweighted and Weighted Sample (in brackets)) 29](#_Toc188371082)

[Supplementary file S4. Sensitivity analyses 30](#_Toc188371083)

[S4.1. Union presence – adjusted model (2-way interaction), excluding union members 30](#_Toc188371084)

[S4.2. Union presence – adjusted model (3-way interaction), excluding union members 33](#_Toc188371085)

[S4.3. Union presence – adjusted model (2-way interaction), excluding public workplaces 39](#_Toc188371086)

[S4.4. Union presence – adjusted model (3-way interaction), excluding public workplaces 42](#_Toc188371087)

[S4.5. Union presence – adjusted model (2-way interaction), GHQ-case – adjusting for trade union membership 47](#_Toc188371088)

[S4.6. Union presence – adjusted model (2-way interaction), GHQ-36 50](#_Toc188371089)

[S4.7. Union membership – adjusted model (2-way interaction), GHQ-36 53](#_Toc188371090)

[S4.8. Union presence – adjusted model (2-way interaction)- Removing Financial situation from confounders’ list 56](#_Toc188371091)

[S4.9. Union presence – adjusted model (2-way interaction)- Unweighted estimates 59](#_Toc188371092)

[S4.10. Marginal means for GHQ-caseness by union presence controlling for union membership and marginal means of GHQ-caseness by union membership controlling for union presence 62](#_Toc188371093)

[Supplementary file S5. Trade union presence and membership and rate of change across Understanding Society (UKHLS) available waves 63](#_Toc188371094)

[Supplementary file S6. STROBE checklist 64](#_Toc188371095)

# Supplementary file 1. Flowchart of study participants

## Union presence flowchart


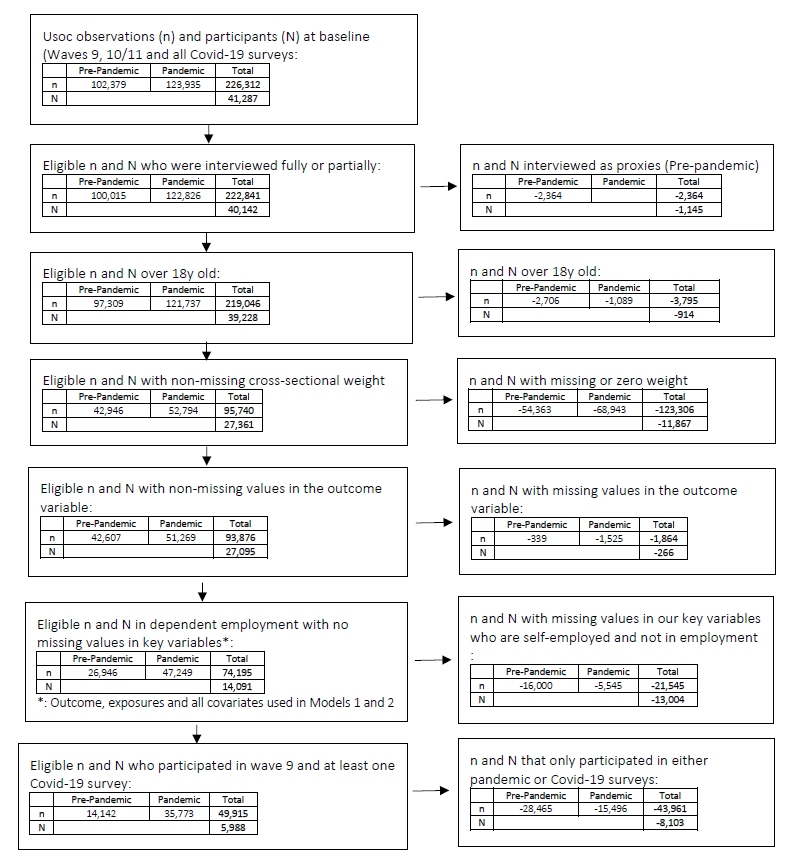


## Union membership flowchart


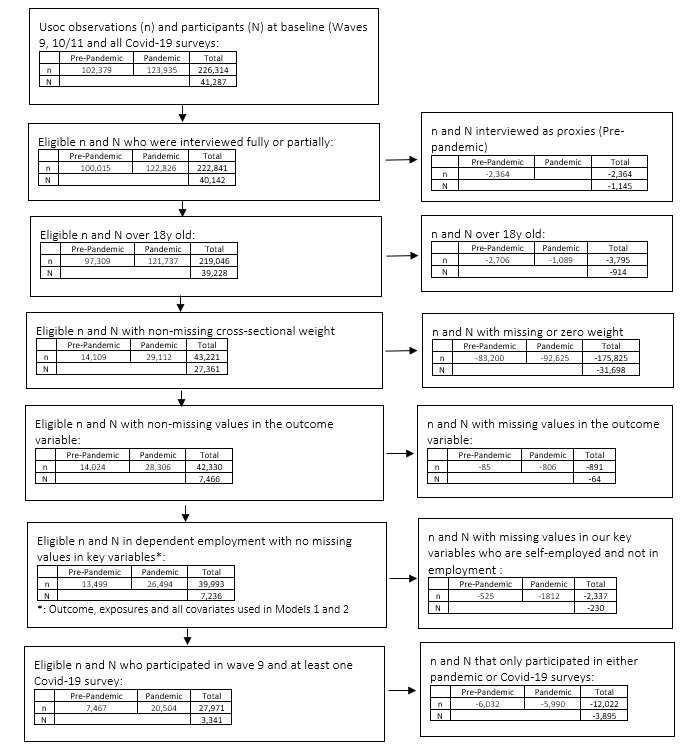


## Testing the stability assumption (Trade Union presence and Trade union membership variables)

Trade union presence and membership variables are asked every two waves and therefore data exist only in waves 2,4,6,8 and 10. Using a sample that includes only these waves we first calculated a variable that shows the change in non-missing values of the trade union presence and membership variables between two consecutive waves. Then we cross-tabulated it over the original variable, again excluding all its missing values. We found that respondents in unionised workplaces and non-unionised workplaces have a very high and also very similar overall stability of 87% and 86%, respectively. For trade union members the equivalent percentages are 93% and 84% for trade union members and non-members, respectively (Tables S3 and S4).

### Table S1:Transitions between trade union presence values (Yes :Unionised workplace, No: Non-unionised workplace)

|  | Frequency | Percentage |
| --- | --- | --- |
| Stable between two waves (non-unionised workplaces) - From No to No | 21,961 | 85.81 |
| Transition from a Unionised to a non-unionised workplace – From Yes to No | 3,632 | 14.19 |
| **Total (non-unionised workplaces)** | **25,593** | **100.00** |
| Stable between two waves (unionised workplaces) - From Yes to Yes | 23,408 | 86.8 |
| Transition from a non-Unionised to a unionised workplace – From No to Yes | 3,561 | 13.20 |
| **Total (unionised workplaces)** | **26,969** | **100.00** |

### Table S2:Transitions between trade union membership values (Yes :member, No: Non-member)

|  | Frequency | Percentage |
| --- | --- | --- |
| Stable between two waves (non-member) - From No to No | 7,104 | 84.36 |
| Transition from member to a non-member – From Yes to No | 1,317 | 15.64 |
| **Total (non-member )** | **8,421** | **100.00** |
| Stable between two waves (member) - From Yes to Yes | 13,884 | 92.94 |
| Transition from a non-member to member – From No to Yes | 1,054 | 7.06 |
| **Total (member)** | **14,938** | **100.00** |

# Supplementary file S2 – Main estimates

## Union presence – unadjusted model

|  |  | **Unadjusted model (weighted)** | | | | | |
| --- | --- | --- | --- | --- | --- | --- | --- |
|  |  | **OR** | **std. err.** | **z** | **P>z** | **[95% conf.** | **interval]** |
| **During pandemic: 1** | | **1.31** | **0.14** | **2.47** | **0.01** | **1.06** | **1.62** |
| **Union presence: yes** | | **1.21** | **0.10** | **2.27** | **0.02** | **1.03** | **1.43** |
| **During pandemic: 1 # union presence: yes** | | **0.86** | **0.08** | **-1.61** | **0.11** | **0.71** | **1.03** |
| Wave Number: | |  |  |  |  |  |  |
|  | Wave 10 | 0.90 | 0.07 | -1.36 | 0.17 | 0.77 | 1.05 |
|  | Wave 11 | 1.19 | 0.10 | 2.07 | 0.04 | 1.01 | 1.41 |
|  | **COVID-19 sweeps** |  |  |  |  |  |  |
|  | Apr-20 | 2.34 | 0.21 | 9.46 | 0.00 | 1.96 | 2.79 |
|  | May-20 | 1.85 | 0.17 | 6.76 | 0.00 | 1.55 | 2.21 |
|  | Jun-20 | 1.63 | 0.16 | 5.00 | 0.00 | 1.34 | 1.97 |
|  | Jul-20 | 0.99 | 0.09 | -0.14 | 0.89 | 0.82 | 1.18 |
|  | Sep-20 | 1.06 | 0.10 | 0.62 | 0.53 | 0.88 | 1.29 |
|  | Nov-20 | 1.85 | 0.19 | 6.11 | 0.00 | 1.52 | 2.25 |
|  | Jan-21 | 1.81 | 0.17 | 6.24 | 0.00 | 1.50 | 2.18 |
|  | Mar-21 | 1.25 | 0.12 | 2.30 | 0.02 | 1.03 | 1.50 |
|  | Sep-21 | 1.00 | (omitted) |  |  |  |  |
| Constant | | 0.08 | 0.01 | -30.08 | 0.00 | 0.06 | 0.09 |

Note: Observations=49,915; Individuals=5,988 (Minimum=1; Average= 8.3; Maximum=12). **Exposure is in bold letters**. ‘#’ indicates interaction effects

## Union presence – adjusted model (2-way interaction)

|  |  | **Adjusted model (weighted)** | | | | | |
| --- | --- | --- | --- | --- | --- | --- | --- |
|  |  | **OR** | **std. err.** | **z** | **P>z** | **[95% conf.** | **interval]** |
| Sex |  | 2.16 | 0.15 | 11.42 | 0.00 | 1.89 | 2.47 |
| Age |  | 1.01 | 0.02 | 0.32 | 0.75 | 0.97 | 1.04 |
| Age_square | | 1.00 | 0.00 | -1.57 | 0.12 | 1.00 | 1.00 |
| Ethnicity | | 0.90 | 0.11 | -0.85 | 0.40 | 0.72 | 1.14 |
| **During pandemic: 1** | | **1.45** | **0.16** | **3.34** | **0.00** | **1.17** | **1.80** |
| **Union presence: yes** | | **1.11** | **0.10** | **1.18** | **0.24** | **0.93** | **1.33** |
| **During pandemic: 1 # Union presence: yes** | | **0.89** | **0.09** | **-1.23** | **0.22** | **0.73** | **1.07** |
| Industry: | |  |  |  |  |  |  |
|  | Mining, Energy and Water Supply | 1.13 | 0.29 | 0.47 | 0.64 | 0.68 | 1.86 |
|  | Manufacturing | 0.66 | 0.13 | -2.16 | 0.03 | 0.45 | 0.96 |
|  | Construction | 0.68 | 0.15 | -1.75 | 0.08 | 0.44 | 1.05 |
|  | Wholesale and Retail Trade Motor Repair | 0.85 | 0.16 | -0.90 | 0.37 | 0.59 | 1.22 |
|  | Transportation and Storage | 0.68 | 0.15 | -1.80 | 0.07 | 0.44 | 1.04 |
|  | Information and Communication | 0.85 | 0.18 | -0.77 | 0.44 | 0.57 | 1.28 |
|  | Financial and Insurance Activities | 0.64 | 0.14 | -2.03 | 0.04 | 0.42 | 0.98 |
|  | Real Estate Activities | 0.96 | 0.27 | -0.16 | 0.88 | 0.55 | 1.65 |
|  | Professional Scientific and Technical | 0.79 | 0.15 | -1.24 | 0.21 | 0.55 | 1.14 |
|  | Administrative and Support Services | 0.62 | 0.13 | -2.32 | 0.02 | 0.41 | 0.93 |
|  | Public Administration and Defence | 0.75 | 0.14 | -1.54 | 0.12 | 0.52 | 1.08 |
|  | Education | 0.78 | 0.14 | -1.36 | 0.17 | 0.55 | 1.11 |
|  | Human Health and Social Work Activities | 0.81 | 0.14 | -1.21 | 0.23 | 0.57 | 1.14 |
|  | Other Services | 0.67 | 0.13 | -2.12 | 0.03 | 0.47 | 0.97 |
| Country: | |  |  |  |  |  |  |
|  | Wales | 1.24 | 0.16 | 1.70 | 0.09 | 0.97 | 1.59 |
|  | Scotland | 0.98 | 0.11 | -0.17 | 0.86 | 0.79 | 1.22 |
|  | Northern Ireland | 0.87 | 0.13 | -0.92 | 0.36 | 0.65 | 1.17 |
| Highest level of education: | |  |  |  |  |  |  |
|  | A-Level | 0.73 | 0.06 | -4.01 | 0.00 | 0.62 | 0.85 |
|  | GCSE | 0.75 | 0.07 | -3.20 | 0.00 | 0.63 | 0.90 |
|  | Other | 0.55 | 0.09 | -3.50 | 0.00 | 0.39 | 0.77 |
|  | None | 0.37 | 0.12 | -3.19 | 0.00 | 0.20 | 0.68 |
| Self-reported financial conditions: bad | | 2.44 | 0.16 | 13.45 | 0.00 | 2.14 | 2.78 |
| Health condition: yes | | 2.09 | 0.14 | 11.25 | 0.00 | 1.84 | 2.38 |
| Company size: | |  |  |  |  |  |  |
|  | 25-199 | 0.89 | 0.07 | -1.46 | 0.14 | 0.76 | 1.04 |
|  | More than 200 | 0.95 | 0.07 | -0.64 | 0.52 | 0.81 | 1.11 |
| Wave Number | |  |  |  |  |  |  |
|  | Wave 10 | 0.91 | 0.07 | -1.23 | 0.22 | 0.78 | 1.06 |
|  | Wave 11 | 1.22 | 0.11 | 2.32 | 0.02 | 1.03 | 1.45 |
|  | **COVID-19 sweeps** |  |  |  |  |  |  |
|  | Apr-20 | 2.29 | 0.20 | 9.22 | 0.00 | 1.92 | 2.73 |
|  | May-20 | 1.81 | 0.17 | 6.47 | 0.00 | 1.51 | 2.16 |
|  | Jun-20 | 1.62 | 0.16 | 4.98 | 0.00 | 1.34 | 1.96 |
|  | Jul-20 | 0.96 | 0.09 | -0.44 | 0.66 | 0.80 | 1.15 |
|  | Sep-20 | 1.04 | 0.10 | 0.42 | 0.68 | 0.86 | 1.26 |
|  | Nov-20 | 1.81 | 0.18 | 5.82 | 0.00 | 1.48 | 2.21 |
|  | Jan-21 | 1.78 | 0.17 | 5.99 | 0.00 | 1.47 | 2.15 |
|  | Mar-21 | 1.27 | 0.12 | 2.43 | 0.02 | 1.05 | 1.54 |
|  | Sep-21 | 1.00 | (omitted) |  |  |  |  |
| Constant | | 0.04 | 0.02 | -7.06 | 0.00 | 0.02 | 0.10 |

Note: Observations=49,915; Individuals=5,988 (Minimum=1; Average= 8.3; Maximum=12). **Exposure is in bold letters.** Reference categories: gender (ref.: female), age, age square, ethnicity (ref.: white), Interaction : ref (pre-pandemic-No presence of trade union), industrial group (ref.: hotel and accommodation), UK country of residence (ref.: England), education (ref.: university degree), financial situation (ref.: Good financial situation), health condition (ref.: Not having longstanding illness or impairment) and size of workplace (ref.:1 to 34 employees), ‘#’ indicates interaction effects

## Union presence – adjusted model (3-way interaction)

|  |  | **Adjusted model (weighted) -- three-ways interaction** | | | | | |
| --- | --- | --- | --- | --- | --- | --- | --- |
|  |  | **OR** | **std. err.** | **z** | **P>z** | **[95% conf.** | **interval]** |
| Sex |  | 2.15 | 0.15 | 11.27 | 0.00 | 1.88 | 2.45 |
| Age |  | 1.01 | 0.02 | 0.32 | 0.75 | 0.97 | 1.04 |
| Age square | | 1.00 | 0.00 | -1.59 | 0.11 | 1.00 | 1.00 |
| Ethnicity | | 0.90 | 0.11 | -0.90 | 0.37 | 0.71 | 1.13 |
| **During pandemic: 1# Industry:** |  |  |  |  |  |  |  |
|  | **Pre-Pandemic-#Mining, Energy and Water Supply** | 2.81 | 1.57 | 1.85 | 0.07 | 0.94 | 8.41 |
|  | **Post-Pandemic-#Mining, Energy and Water Supply** | 2.07 | 0.64 | 2.37 | 0.02 | 1.13 | 3.79 |
|  | **Pre-Pandemic-#Manufacturing** | 0.82 | 0.20 | -0.81 | 0.42 | 0.51 | 1.32 |
|  | **Post-Pandemic-#Manufacturing** | 1.29 | 0.31 | 1.06 | 0.29 | 0.81 | 2.05 |
|  | **Pre-Pandemic-#Construction** | 0.67 | 0.21 | -1.25 | 0.21 | 0.36 | 1.25 |
|  | **Post-Pandemic-#Construction** | 1.11 | 0.29 | 0.41 | 0.68 | 0.67 | 1.86 |
|  | **Pre-Pandemic-#Wholesale and Retail Trade Motor Repair** | 1.04 | 0.26 | 0.14 | 0.89 | 0.64 | 1.69 |
|  | **Post-Pandemic-#Wholesale and Retail Trade Motor Repair** | 1.53 | 0.37 | 1.75 | 0.08 | 0.95 | 2.46 |
|  | **Pre-Pandemic-#Transportation and Storage** | 0.92 | 0.34 | -0.23 | 0.82 | 0.45 | 1.88 |
|  | **Post-Pandemic-#Transportation and Storage** | 0.85 | 0.28 | -0.49 | 0.63 | 0.44 | 1.63 |
|  | **Pre-Pandemic-#Accommodation and Food Services** | 1.59 | 0.49 | 1.51 | 0.13 | 0.87 | 2.91 |
|  | **Post-Pandemic-#Accommodation and Food Services** | 1.65 | 0.48 | 1.71 | 0.09 | 0.93 | 2.92 |
|  | **Pre-Pandemic-#Information and Communication** | 0.71 | 0.25 | -0.96 | 0.34 | 0.36 | 1.42 |
|  | **Post-Pandemic-#Information and Communication** | 1.59 | 0.37 | 2.01 | 0.05 | 1.01 | 2.50 |
|  | **Pre-Pandemic-#Financial and Insurance Activities** | 1.43 | 0.43 | 1.20 | 0.23 | 0.80 | 2.56 |
|  | **Post-Pandemic-#Financial and Insurance Activities** | 1.03 | 0.26 | 0.12 | 0.90 | 0.63 | 1.70 |
|  | **Pre-Pandemic-#Real Estate Activities** | 0.81 | 0.44 | -0.39 | 0.70 | 0.28 | 2.33 |
|  | **Post-Pandemic-#Real Estate Activities** | 1.54 | 0.61 | 1.10 | 0.27 | 0.71 | 3.35 |
|  | **Pre-Pandemic-#Professional Scientific and Technical** | 0.83 | 0.19 | -0.79 | 0.43 | 0.53 | 1.30 |
|  | **Post-Pandemic-#Professional Scientific and Technical** | 1.37 | 0.30 | 1.44 | 0.15 | 0.89 | 2.09 |
|  | **Pre-Pandemic-#Administrative and Support Services** | 0.58 | 0.20 | -1.59 | 0.11 | 0.29 | 1.14 |
|  | **Post-Pandemic-#Administrative and Support Services** | 1.05 | 0.28 | 0.19 | 0.85 | 0.63 | 1.76 |
|  | **Pre-Pandemic-#Public Administration and Defence** | 0.43 | 0.26 | -1.38 | 0.17 | 0.13 | 1.42 |
|  | **Post-Pandemic-#Public Administration and Defence** | 1.07 | 0.33 | 0.21 | 0.83 | 0.58 | 1.96 |
|  | **Pre-Pandemic-#Education** | 0.51 | 0.17 | -2.03 | 0.04 | 0.27 | 0.98 |
|  | **Post-Pandemic-#Education** | 1.47 | 0.35 | 1.61 | 0.11 | 0.92 | 2.34 |
|  | **Post-Pandemic-#Human Health and Social Work Activities** | 1.22 | 0.25 | 0.99 | 0.32 | 0.82 | 1.81 |
|  | **Pre-Pandemic-#Other Services** | 1.15 | 0.36 | 0.45 | 0.65 | 0.62 | 2.14 |
|  | **Post-Pandemic-#Other Services** | 1.18 | 0.25 | 0.79 | 0.43 | 0.78 | 1.79 |
| **Union presence: yes** | | 1.10 | 0.21 | 0.51 | 0.61 | 0.76 | 1.60 |
| **During pandemic: 1 # Industry # Union presence** | |  |  |  |  |  |  |
|  | **Pre-Pandemic-#Mining, Energy and Water Supply#Yes** | 0.48 | 0.35 | -1.02 | 0.31 | 0.11 | 1.98 |
|  | **Post-Pandemic-#Mining, Energy and Water Supply#Yes** | 0.57 | 0.28 | -1.15 | 0.25 | 0.22 | 1.48 |
|  | **Pre-Pandemic-#Manufacturing#Yes** | 0.60 | 0.23 | -1.33 | 0.18 | 0.28 | 1.28 |
|  | **Post-Pandemic-#Manufacturing#Yes** | 0.56 | 0.20 | -1.66 | 0.10 | 0.28 | 1.11 |
|  | **Pre-Pandemic-#Construction#Yes** | 2.09 | 1.16 | 1.32 | 0.19 | 0.70 | 6.22 |
|  | **Post-Pandemic-#Construction#Yes** | 0.99 | 0.41 | -0.02 | 0.99 | 0.45 | 2.22 |
|  | **Pre-Pandemic-#Wholesale and Retail Trade Motor Repair#Yes** | 0.98 | 0.33 | -0.06 | 0.95 | 0.51 | 1.90 |
|  | **Post-Pandemic-#Wholesale and Retail Trade Motor Repair#Yes** | 0.71 | 0.21 | -1.14 | 0.25 | 0.39 | 1.28 |
|  | **Pre-Pandemic-#Transportation and Storage#Yes** | 0.76 | 0.34 | -0.61 | 0.54 | 0.32 | 1.82 |
|  | **Post-Pandemic-#Transportation and Storage#Yes** | 1.48 | 0.59 | 0.99 | 0.32 | 0.68 | 3.25 |
|  | **Pre-Pandemic-#Accommodation and Food Services#Yes** | 0.30 | 0.19 | -1.91 | 0.06 | 0.09 | 1.03 |
|  | **Post-Pandemic-#Accommodation and Food Services#Yes** | 0.89 | 0.39 | -0.27 | 0.79 | 0.38 | 2.10 |
|  | **Pre-Pandemic-#Information and Communication#Yes** | 3.08 | 1.85 | 1.86 | 0.06 | 0.94 | 10.02 |
|  | **Post-Pandemic-#Information and Communication#Yes** | 0.72 | 0.25 | -0.94 | 0.35 | 0.36 | 1.43 |
|  | **Pre-Pandemic-#Financial and Insurance Activities#Yes** | 0.54 | 0.24 | -1.38 | 0.17 | 0.22 | 1.30 |
|  | **Post-Pandemic-#Financial and Insurance Activities#Yes** | 0.74 | 0.26 | -0.86 | 0.39 | 0.37 | 1.47 |
|  | **Pre-Pandemic-#Real Estate Activities#Yes** | 4.32 | 3.03 | 2.08 | 0.04 | 1.09 | 17.09 |
|  | **Post-Pandemic-#Real Estate Activities#Yes** | 0.48 | 0.31 | -1.15 | 0.25 | 0.13 | 1.68 |
|  | **Pre-Pandemic-#Professional Scientific and Technical#Yes** | 0.95 | 0.36 | -0.14 | 0.89 | 0.45 | 2.01 |
|  | **Post-Pandemic-#Professional Scientific and Technical#Yes** | 1.00 | 0.27 | -0.01 | 0.99 | 0.59 | 1.69 |
|  | **Pre-Pandemic-#Administrative and Support Services#Yes** | 1.12 | 0.69 | 0.18 | 0.86 | 0.33 | 3.74 |
|  | **Post-Pandemic-#Administrative and Support Services#Yes** | 0.99 | 0.32 | -0.03 | 0.97 | 0.52 | 1.88 |
|  | **Pre-Pandemic-#Public Administration and Defence#Yes** | 2.11 | 1.33 | 1.19 | 0.23 | 0.62 | 7.23 |
|  | **Post-Pandemic-#Public Administration and Defence#Yes** | 1.16 | 0.39 | 0.45 | 0.66 | 0.60 | 2.23 |
|  | **Pre-Pandemic-#Education#Yes** | 1.74 | 0.62 | 1.56 | 0.12 | 0.87 | 3.51 |
|  | **Post-Pandemic-#Education#Yes** | 0.86 | 0.22 | -0.58 | 0.56 | 0.52 | 1.43 |
|  | **Post-Pandemic-#Human Health and Social Work Activities#Yes** | 1.09 | 0.25 | 0.39 | 0.69 | 0.70 | 1.70 |
|  | **Pre-Pandemic-#Other Services #Yes** | 0.67 | 0.31 | -0.85 | 0.40 | 0.27 | 1.68 |
|  | **Post-Pandemic-#Other Services #Yes** | 0.80 | 0.22 | -0.81 | 0.42 | 0.47 | 1.36 |
| Country: | |  |  |  |  |  |  |
|  | Wales | 1.25 | 0.16 | 1.76 | 0.08 | 0.97 | 1.61 |
|  | Scotland | 0.98 | 0.11 | -0.22 | 0.83 | 0.78 | 1.22 |
|  | Northern Ireland | 0.87 | 0.13 | -0.94 | 0.35 | 0.65 | 1.16 |
| Highest level of education: | |  |  |  |  |  |  |
|  | A-Level | 0.74 | 0.06 | -3.81 | 0.00 | 0.63 | 0.86 |
|  | GCSE | 0.77 | 0.07 | -2.97 | 0.00 | 0.64 | 0.91 |
|  | Other | 0.55 | 0.10 | -3.45 | 0.00 | 0.39 | 0.77 |
|  | None | 0.38 | 0.12 | -3.07 | 0.00 | 0.20 | 0.70 |
| Self-reported financial conditions: bad | | 2.45 | 0.16 | 13.45 | 0.00 | 2.15 | 2.79 |
| Health condition: yes | | 2.08 | 0.14 | 11.18 | 0.00 | 1.83 | 2.37 |
| Company size: | |  |  |  |  |  |  |
|  | 25-199 | 0.90 | 0.07 | -1.38 | 0.17 | 0.77 | 1.05 |
|  | More than 200 | 0.96 | 0.08 | -0.55 | 0.59 | 0.82 | 1.12 |
| Wave Number | |  |  |  |  |  |  |
|  | Wave 10 | 0.91 | 0.07 | -1.26 | 0.21 | 0.78 | 1.06 |
|  | Wave 11 | 1.24 | 0.11 | 2.51 | 0.01 | 1.05 | 1.47 |
|  | **COVID-19 sweeps** |  |  |  |  |  |  |
|  | Apr-20 | 2.29 | 0.21 | 9.25 | 0.00 | 1.92 | 2.73 |
|  | May-20 | 1.81 | 0.17 | 6.50 | 0.00 | 1.51 | 2.17 |
|  | Jun-20 | 1.62 | 0.16 | 5.00 | 0.00 | 1.34 | 1.96 |
|  | Jul-20 | 0.96 | 0.09 | -0.46 | 0.64 | 0.80 | 1.15 |
|  | Sep-20 | 1.04 | 0.10 | 0.38 | 0.71 | 0.86 | 1.26 |
|  | Nov-20 | 1.81 | 0.18 | 5.81 | 0.00 | 1.48 | 2.21 |
|  | Jan-21 | 1.77 | 0.17 | 5.95 | 0.00 | 1.47 | 2.14 |
|  | Mar-21 | 1.27 | 0.12 | 2.44 | 0.02 | 1.05 | 1.54 |
|  | Sep-21 | 1.00 | (omitted) |  |  |  |  |
| Constant | | 0.04 | 0.02 | -7.22 | 0.00 | 0.01 | 0.09 |

Note: Observations=49,915; Individuals=5,988 (Minimum=1; Average= 8.3; Maximum=12). **Exposure is in bold letters.** Reference categories: gender (ref.: female), age, age square, ethnicity (ref.: white), Interaction : ref (pre-pandemic-No presence of trade union-Hotel and Accommodation Industry ), UK country of residence (ref.: England), education (ref.: university degree), financial situation (ref.: Good financial situation), health condition (ref.: Not having longstanding illness or impairment) and size of workplace (ref.:1 to 34 employees),‘#’ indicates interaction effects.

## Union membership – unadjusted model

|  |  | **Unadjusted model (weighted)** | | | | | |
| --- | --- | --- | --- | --- | --- | --- | --- |
|  |  | **OR** | **std. err.** | **z** | **P>z** | **[95% conf.** | **interval]** |
| During pandemic: 1 | | 1.24 | 0.17 | 1.57 | 0.12 | 0.95 | 1.63 |
| **Union membership: yes** | | 1.24 | 0.15 | 1.85 | 0.06 | 0.99 | 1.56 |
| **During pandemic: 1 # union membership: yes** | | 0.85 | 0.11 | -1.27 | 0.21 | 0.66 | 1.09 |
| **Wave Number:** | |  |  |  |  |  |  |
|  | Wave 10 | 0.88 | 0.09 | -1.24 | 0.22 | 0.71 | 1.08 |
|  | Wave 11 | 1.32 | 0.16 | 2.35 | 0.02 | 1.05 | 1.67 |
|  | **COVID-19 sweeps** |  |  |  |  |  |  |
|  | Apr-20 | 2.54 | 0.28 | 8.44 | 0.00 | 2.05 | 3.15 |
|  | May-20 | 1.77 | 0.20 | 4.94 | 0.00 | 1.41 | 2.22 |
|  | Jun-20 | 1.49 | 0.18 | 3.25 | 0.00 | 1.17 | 1.90 |
|  | Jul-20 | 0.87 | 0.10 | -1.18 | 0.24 | 0.69 | 1.10 |
|  | Sep-20 | 1.12 | 0.13 | 0.92 | 0.36 | 0.88 | 1.41 |
|  | Nov-20 | 1.97 | 0.24 | 5.53 | 0.00 | 1.55 | 2.50 |
|  | Jan-21 | 2.22 | 0.25 | 7.05 | 0.00 | 1.78 | 2.77 |
|  | Mar-21 | 1.24 | 0.15 | 1.75 | 0.08 | 0.97 | 1.58 |
|  | Sep-21 | 1.00 | (omitted) |  |  |  |  |
| Constant | | 0.08 | 0.01 | -21.86 | 0.00 | 0.07 | 0.10 |

Note: Observations=27,971; Individuals=3,341 (Minimum=1; Average= 8.4; Maximum=12). **Exposure is in bold letters**,‘#’ indicates interaction effects

## Union membership – adjusted model (2-ways interaction)

|  |  | **Adjusted model (weighted)** | | | | | |
| --- | --- | --- | --- | --- | --- | --- | --- |
|  |  | **OR** | **std. err.** | **z** | **P>z** | **[95% conf.** | **interval]** |
| Sex |  | 2.08 | 0.20 | 7.81 | 0.00 | 1.73 | 2.50 |
| Age |  | 1.02 | 0.03 | 0.77 | 0.44 | 0.97 | 1.08 |
| Age_square | | 1.00 | 0.00 | -1.32 | 0.19 | 1.00 | 1.00 |
| Ethnicity | | 0.82 | 0.12 | -1.40 | 0.16 | 0.61 | 1.08 |
| **During pandemic: 1** | | **1.40** | **0.19** | **2.43** | **0.02** | **1.07** | **1.83** |
| **Union membership: yes** | | **1.17** | **0.14** | **1.37** | **0.17** | **0.93** | **1.47** |
| **During pandemic: 1 # Union membership: yes** | | **0.84** | **0.11** | **-1.35** | **0.18** | **0.66** | **1.08** |
| Industry: | |  |  |  |  |  |  |
|  | Mining, Energy and Water Supply | 1.08 | 0.44 | 0.20 | 0.84 | 0.49 | 2.42 |
|  | Manufacturing | 0.62 | 0.20 | -1.46 | 0.14 | 0.33 | 1.18 |
|  | Construction | 1.08 | 0.42 | 0.21 | 0.84 | 0.51 | 2.32 |
|  | Wholesale and Retail Trade Motor Repair | 1.07 | 0.32 | 0.21 | 0.83 | 0.59 | 1.93 |
|  | Transportation and Storage | 1.02 | 0.31 | 0.08 | 0.94 | 0.56 | 1.86 |
|  | Information and Communication | 1.18 | 0.40 | 0.49 | 0.63 | 0.61 | 2.30 |
|  | Financial and Insurance Activities | 0.63 | 0.21 | -1.37 | 0.17 | 0.32 | 1.22 |
|  | Real Estate Activities | 1.35 | 0.51 | 0.80 | 0.43 | 0.65 | 2.82 |
|  | Professional Scientific and Technical | 1.16 | 0.34 | 0.51 | 0.61 | 0.65 | 2.06 |
|  | Administrative and Support Services | 0.82 | 0.26 | -0.63 | 0.53 | 0.44 | 1.52 |
|  | Public Administration and Defence | 1.00 | 0.27 | 0.00 | 1.00 | 0.58 | 1.71 |
|  | Education | 1.05 | 0.28 | 0.19 | 0.85 | 0.62 | 1.77 |
|  | Human Health and Social Work Activities | 1.11 | 0.29 | 0.40 | 0.69 | 0.66 | 1.87 |
|  | Other Services | 0.80 | 0.23 | -0.78 | 0.44 | 0.45 | 1.41 |
| Country: | |  |  |  |  |  |  |
|  | Wales | 1.04 | 0.17 | 0.26 | 0.79 | 0.76 | 1.43 |
|  | Scotland | 0.94 | 0.12 | -0.49 | 0.62 | 0.73 | 1.21 |
|  | Northern Ireland | 0.89 | 0.16 | -0.66 | 0.51 | 0.63 | 1.26 |
| Highest level of education: | |  |  |  |  |  |  |
|  | A-Level | 0.75 | 0.08 | -2.66 | 0.01 | 0.61 | 0.93 |
|  | GCSE | 0.64 | 0.08 | -3.58 | 0.00 | 0.50 | 0.82 |
|  | Other | 0.45 | 0.11 | -3.34 | 0.00 | 0.28 | 0.72 |
|  | None | 0.42 | 0.18 | -2.00 | 0.05 | 0.18 | 0.98 |
| Self-reported financial conditions: bad | | 2.74 | 0.22 | 12.30 | 0.00 | 2.33 | 3.22 |
| Health condition: yes | | 1.94 | 0.17 | 7.57 | 0.00 | 1.63 | 2.30 |
| Company size: | |  |  |  |  |  |  |
|  | 25-199 | 0.95 | 0.11 | -0.45 | 0.65 | 0.75 | 1.19 |
|  | More than 200 | 1.05 | 0.12 | 0.48 | 0.63 | 0.85 | 1.31 |
| Wave Number | |  |  |  |  |  |  |
|  | Wave 10 | 0.89 | 0.09 | -1.12 | 0.26 | 0.72 | 1.09 |
|  | Wave 11 | 1.35 | 0.16 | 2.51 | 0.01 | 1.07 | 1.70 |
|  | **COVID-19 sweeps** |  |  |  |  |  |  |
|  | Apr-20 | 2.57 | 0.28 | 8.63 | 0.00 | 2.07 | 3.18 |
|  | May-20 | 1.78 | 0.20 | 5.02 | 0.00 | 1.42 | 2.23 |
|  | Jun-20 | 1.55 | 0.19 | 3.55 | 0.00 | 1.22 | 1.98 |
|  | Jul-20 | 0.87 | 0.10 | -1.19 | 0.23 | 0.69 | 1.10 |
|  | Sep-20 | 1.13 | 0.13 | 0.99 | 0.32 | 0.89 | 1.42 |
|  | Nov-20 | 1.95 | 0.24 | 5.35 | 0.00 | 1.53 | 2.50 |
|  | Jan-21 | 2.23 | 0.25 | 7.02 | 0.00 | 1.78 | 2.79 |
|  | Mar-21 | 1.27 | 0.16 | 1.93 | 0.05 | 1.00 | 1.62 |
|  | Sep-21 | 1.00 | (omitted) |  |  |  |  |
| Constant | | 0.02 | 0.01 | -5.65 | 0.00 | 0.01 | 0.08 |

Note: Observations=27,971; Individuals=3,341 (Minimum=1; Average= 8.4; Maximum=12). **Exposure is in bold letters**. Reference categories: gender (ref.: female), age, age square, ethnicity (ref.: white), Interaction : ref (pre-pandemic-No presence of trade union), industrial group (ref.: hotel and accommodation), UK country of residence (ref.: England), education (ref.: university degree), financial situation (ref.: Good financial situation), health condition (ref.: Not having longstanding illness or impairment) and size of workplace (ref.:1 to 34 employees), ‘#’ indicates interaction effects

## Union membership – adjusted model (3-ways interaction)

|  |  | **Adjusted model (weighted) -- three-ways interaction** | | | | | |
| --- | --- | --- | --- | --- | --- | --- | --- |
|  |  | **OR** | **std. err.** | **z** | **P>z** | **[95% conf.** | **interval]** |
| Sex |  | 2.11 | 0.20 | 7.90 | 0.00 | 1.75 | 2.54 |
| Age |  | 1.02 | 0.03 | 0.74 | 0.46 | 0.97 | 1.08 |
| Age square | | 1.00 | 0.00 | -1.29 | 0.20 | 1.00 | 1.00 |
| Ethnicity | | 0.81 | 0.12 | -1.44 | 0.15 | 0.61 | 1.08 |
| **During pandemic: 1 # Industry:** |  |  |  |  |  |  |  |
|  | **Pre-Pandemic-#Mining, Energy and Water Supply** | 0.98 | 0.79 | -0.02 | 0.98 | 0.20 | 4.77 |
|  | **Post-Pandemic-#Mining, Energy and Water Supply** | 1.13 | 0.69 | 0.19 | 0.85 | 0.34 | 3.77 |
|  | **Pre-Pandemic-#Manufacturing** | 0.25 | 0.15 | -2.33 | 0.02 | 0.08 | 0.80 |
|  | **Post-Pandemic-#Manufacturing** | 0.64 | 0.26 | -1.08 | 0.28 | 0.29 | 1.44 |
|  | **Pre-Pandemic-#Construction** | 1.79 | 1.09 | 0.96 | 0.34 | 0.54 | 5.91 |
|  | **Post-Pandemic-#Construction** | 0.88 | 0.39 | -0.30 | 0.76 | 0.37 | 2.08 |
|  | **Pre-Pandemic-#Wholesale and Retail Trade Motor Repair** | 1.12 | 0.43 | 0.29 | 0.78 | 0.52 | 2.38 |
|  | **Post-Pandemic-#Wholesale and Retail Trade Motor Repair** | 1.23 | 0.38 | 0.67 | 0.51 | 0.67 | 2.25 |
|  | **Pre-Pandemic-#Transportation and Storage** | 0.39 | 0.21 | -1.75 | 0.08 | 0.13 | 1.12 |
|  | **Post-Pandemic-#Transportation and Storage** | 0.92 | 0.40 | -0.20 | 0.84 | 0.39 | 2.15 |
|  | **Pre-Pandemic-#Accommodation and Food Services** | 0.71 | 0.44 | -0.56 | 0.58 | 0.21 | 2.41 |
|  | **Post-Pandemic-#Accommodation and Food Services** | 1.38 | 0.51 | 0.87 | 0.38 | 0.67 | 2.87 |
|  | **Pre-Pandemic-#Information and Communication** | 1.30 | 0.77 | 0.44 | 0.66 | 0.40 | 4.16 |
|  | **Post-Pandemic-#Information and Communication** | 1.57 | 0.61 | 1.17 | 0.24 | 0.74 | 3.35 |
|  | **Pre-Pandemic-#Financial and Insurance Activities** | 0.38 | 0.16 | -2.28 | 0.02 | 0.16 | 0.87 |
|  | **Post-Pandemic-#Financial and Insurance Activities** | 0.75 | 0.25 | -0.84 | 0.40 | 0.39 | 1.46 |
|  | **Pre-Pandemic-#Real Estate Activities** | 2.50 | 1.29 | 1.78 | 0.08 | 0.91 | 6.89 |
|  | **Post-Pandemic-#Real Estate Activities** | 0.96 | 0.58 | -0.07 | 0.94 | 0.29 | 3.16 |
|  | **Pre-Pandemic-#Professional Scientific and Technical** | 0.76 | 0.35 | -0.58 | 0.56 | 0.31 | 1.90 |
|  | **Post-Pandemic-#Professional Scientific and Technical** | 1.53 | 0.44 | 1.48 | 0.14 | 0.87 | 2.67 |
|  | **Pre-Pandemic-#Administrative and Support Services** | 0.24 | 0.17 | -2.03 | 0.04 | 0.06 | 0.95 |
|  | **Post-Pandemic-#Administrative and Support Services** | 1.03 | 0.31 | 0.09 | 0.93 | 0.57 | 1.85 |
|  | **Pre-Pandemic-#Public Administration and Defence** | 0.78 | 0.22 | -0.89 | 0.37 | 0.45 | 1.35 |
|  | **Post-Pandemic-#Public Administration and Defence** | 0.95 | 0.24 | -0.19 | 0.85 | 0.58 | 1.57 |
|  | **Pre-Pandemic-#Education** | 0.85 | 0.22 | -0.63 | 0.53 | 0.51 | 1.41 |
|  | **Post-Pandemic-#Education** | 1.15 | 0.27 | 0.57 | 0.57 | 0.72 | 1.83 |
|  | **Post-Pandemic-#Human Health and Social Work Activities** | 1.25 | 0.28 | 0.96 | 0.34 | 0.80 | 1.95 |
|  | **Pre-Pandemic-#Other Services** | 0.26 | 0.12 | -3.01 | 0.00 | 0.11 | 0.62 |
|  | **Post-Pandemic-#Other Services** | 0.74 | 0.23 | -0.95 | 0.34 | 0.41 | 1.37 |
| **Union membership: yes** | | 0.89 | 0.20 | -0.55 | 0.58 | 0.57 | 1.37 |
| **During pandemic: 1 # Industry # Union membership** | |  |  |  |  |  |  |
|  | **Pre-Pandemic-#Mining, Energy and Water Supply#Yes** | 1.75 | 1.72 | 0.57 | 0.57 | 0.26 | 12.00 |
|  | **Post-Pandemic-#Mining, Energy and Water Supply#Yes** | 1.04 | 0.74 | 0.05 | 0.96 | 0.26 | 4.17 |
|  | **Pre-Pandemic-#Manufacturing#Yes** | 3.19 | 2.18 | 1.69 | 0.09 | 0.83 | 12.19 |
|  | **Post-Pandemic-#Manufacturing#Yes** | 1.36 | 0.70 | 0.60 | 0.55 | 0.50 | 3.74 |
|  | **Pre-Pandemic-#Construction#Yes** | 0.71 | 0.65 | -0.38 | 0.71 | 0.12 | 4.29 |
|  | **Post-Pandemic-#Construction#Yes** | 1.41 | 0.85 | 0.57 | 0.57 | 0.43 | 4.60 |
|  | **Pre-Pandemic-#Wholesale and Retail Trade Motor Repair#Yes** | 0.92 | 0.47 | -0.17 | 0.87 | 0.33 | 2.52 |
|  | **Post-Pandemic-#Wholesale and Retail Trade Motor Repair#Yes** | 0.90 | 0.36 | -0.27 | 0.79 | 0.41 | 1.98 |
|  | **Pre-Pandemic-#Transportation and Storage#Yes** | 2.60 | 1.65 | 1.50 | 0.13 | 0.75 | 9.01 |
|  | **Post-Pandemic-#Transportation and Storage#Yes** | 1.65 | 0.84 | 0.99 | 0.32 | 0.61 | 4.45 |
|  | **Pre-Pandemic-#Accommodation and Food Services#Yes** | 0.07 | 0.09 | -2.13 | 0.03 | 0.01 | 0.80 |
|  | **Post-Pandemic-#Accommodation and Food Services#Yes** | 0.92 | 0.61 | -0.12 | 0.91 | 0.25 | 3.39 |
|  | **Pre-Pandemic-#Information and Communication#Yes** | 2.06 | 1.79 | 0.83 | 0.41 | 0.37 | 11.30 |
|  | **Post-Pandemic-#Information and Communication#Yes** | 0.50 | 0.26 | -1.34 | 0.18 | 0.18 | 1.38 |
|  | **Pre-Pandemic-#Financial and Insurance Activities#Yes** | 3.78 | 2.56 | 1.97 | 0.05 | 1.00 | 14.22 |
|  | **Post-Pandemic-#Financial and Insurance Activities#Yes** | 0.72 | 0.39 | -0.61 | 0.54 | 0.25 | 2.07 |
|  | **Pre-Pandemic-#Real Estate Activities#Yes** | 1.80 | 1.48 | 0.72 | 0.47 | 0.36 | 8.99 |
|  | **Post-Pandemic-#Real Estate Activities#Yes** | 0.51 | 0.38 | -0.91 | 0.36 | 0.12 | 2.17 |
|  | **Pre-Pandemic-#Professional Scientific and Technical#Yes** | 1.13 | 0.72 | 0.19 | 0.85 | 0.32 | 3.97 |
|  | **Post-Pandemic-#Professional Scientific and Technical#Yes** | 0.84 | 0.30 | -0.50 | 0.62 | 0.42 | 1.68 |
|  | **Pre-Pandemic-#Administrative and Support Services#Yes** | 8.34 | 8.07 | 2.19 | 0.03 | 1.25 | 55.62 |
|  | **Post-Pandemic-#Administrative and Support Services#Yes** | 0.74 | 0.31 | -0.72 | 0.47 | 0.33 | 1.67 |
|  | **Pre-Pandemic-#Public Administration and Defence#Yes** | 1.34 | 0.47 | 0.85 | 0.40 | 0.68 | 2.66 |
|  | **Post-Pandemic-#Public Administration and Defence#Yes** | 1.49 | 0.45 | 1.32 | 0.19 | 0.83 | 2.67 |
|  | **Pre-Pandemic-#Education#Yes** | 1.13 | 0.36 | 0.37 | 0.71 | 0.60 | 2.12 |
|  | **Post-Pandemic-#Education#Yes** | 1.17 | 0.33 | 0.57 | 0.57 | 0.68 | 2.02 |
|  | **Post-Pandemic-#Human Health and Social Work Activities#Yes** | 1.07 | 0.29 | 0.25 | 0.80 | 0.63 | 1.80 |
|  | **Pre-Pandemic-#Other Services #Yes** | 8.86 | 5.47 | 3.53 | 0.00 | 2.64 | 29.74 |
|  | **Post-Pandemic-#Other Services #Yes** | 1.54 | 0.57 | 1.16 | 0.25 | 0.74 | 3.17 |
| Country: | |  |  |  |  |  |  |
|  | Wales | 1.04 | 0.17 | 0.26 | 0.79 | 0.76 | 1.44 |
|  | Scotland | 0.95 | 0.13 | -0.36 | 0.72 | 0.74 | 1.23 |
|  | Northern Ireland | 0.90 | 0.16 | -0.60 | 0.55 | 0.63 | 1.28 |
| Highest level of education: | |  |  |  |  |  |  |
|  | A-Level | 0.75 | 0.08 | -2.68 | 0.01 | 0.61 | 0.92 |
|  | GCSE | 0.64 | 0.08 | -3.64 | 0.00 | 0.50 | 0.81 |
|  | Other | 0.45 | 0.11 | -3.34 | 0.00 | 0.28 | 0.72 |
|  | None | 0.42 | 0.19 | -1.96 | 0.05 | 0.17 | 1.00 |
| Self-reported financial conditions: bad | | 2.75 | 0.23 | 12.24 | 0.00 | 2.34 | 3.23 |
| Health condition: yes | | 1.92 | 0.17 | 7.49 | 0.00 | 1.62 | 2.27 |
| Company size: | |  |  |  |  |  |  |
|  | 25-199 | 0.94 | 0.11 | -0.53 | 0.60 | 0.75 | 1.18 |
|  | More than 200 | 1.05 | 0.12 | 0.43 | 0.67 | 0.84 | 1.31 |
| Wave Number | |  |  |  |  |  |  |
|  | Wave 10 | 0.88 | 0.09 | -1.18 | 0.24 | 0.72 | 1.09 |
|  | Wave 11 | 1.35 | 0.16 | 2.55 | 0.01 | 1.07 | 1.70 |
|  | **COVID-19 sweeps** |  |  |  |  |  |  |
|  | Apr-20 | 2.59 | 0.28 | 8.70 | 0.00 | 2.09 | 3.21 |
|  | May-20 | 1.79 | 0.20 | 5.06 | 0.00 | 1.43 | 2.24 |
|  | Jun-20 | 1.55 | 0.19 | 3.52 | 0.00 | 1.21 | 1.97 |
|  | Jul-20 | 0.87 | 0.10 | -1.20 | 0.23 | 0.69 | 1.09 |
|  | Sep-20 | 1.12 | 0.13 | 0.98 | 0.33 | 0.89 | 1.42 |
|  | Nov-20 | 1.95 | 0.24 | 5.31 | 0.00 | 1.52 | 2.49 |
|  | Jan-21 | 2.23 | 0.25 | 7.01 | 0.00 | 1.78 | 2.79 |
|  | Mar-21 | 1.27 | 0.16 | 1.90 | 0.06 | 0.99 | 1.62 |
|  | Sep-21 | 1.00 | (omitted) |  |  |  |  |
| Constant | | 0.03 | 0.02 | -5.33 | 0.00 | 0.01 | 0.10 |

Note: Observations=27,971; Individuals=3,341 (Minimum=1; Average= 8.4; Maximum=12). **Exposure is in bold letters**. Reference categories: gender (ref.: female), age, age square, ethnicity (ref.: white), Interaction : ref (pre-pandemic-No presence of trade union-Hotel and Accommodation Industry ), UK country of residence (ref.: England), education (ref.: university degree), financial situation (ref.: Good financial situation), health condition (ref.: Not having longstanding illness or impairment) and size of workplace (ref.:1 to 34 employees),‘#’ indicates interaction effects.

## Table 2 (bis): Adjusted Models (Model 2, by industry) Marginal Means (MM) and Average Marginal Effects (AVE) for trade Union Presence and trade union membership as exposures

| Model 2 (industries) |  |  | **Trade union presence**  **(Full sample)** | | | | | | | | | | |
| --- | --- | --- | --- | --- | --- | --- | --- | --- | --- | --- | --- | --- | --- |
|  | **Time Period** | **Trade Union** | **MM** | **95 % CI** | | | | **AME** | | **95 % CI** | | |  |
|  |  |  |  | **Lower** | | **Higher** | |  |  | **Lower** | **Higher** | |  |
| Mining, Energy and Water Supply (MEWS) | Pre-pandemic | No | 0.258 | 0.076 | | 0.439 | |  | |  |  | |  |
|  | Pre-pandemic | Yes | 0.162 | 0.048 | | 0.276 | | -0.096 | | -0.307 | 0.116 | |  |
|  | pandemic | No | 0.209 | 0.135 | | 0.283 | |  | |  |  | |  |
|  | pandemic | Yes | 0.148 | 0.064 | | 0.233 | | -0.061 | | -0.172 | 0.050 | |  |
| Manufacturing (MAN) | Pre-pandemic | No | 0.101 | 0.070 | | 0.133 | |  | |  |  | |  |
|  | Pre-pandemic | Yes | 0.070 | 0.031 | | 0.110 | | -0.031 | | -0.077 | 0.015 | |  |
|  | pandemic | No | 0.146 | 0.110 | | 0.182 | |  | |  |  | |  |
|  | pandemic | Yes | 0.098 | 0.057 | | 0.138 | | -0.048 | | -0.103 | 0.007 | |  |
| Construction (CON) | Pre-pandemic | No | 0.085 | 0.044 | | 0.127 | |  | |  |  | |  |
|  | Pre-pandemic | Yes | 0.168 | 0.051 | | 0.285 | | 0.083 | | -0.038 | 0.204 | |  |
|  | pandemic | No | 0.130 | 0.090 | | 0.170 | |  | |  |  | |  |
|  | pandemic | Yes | 0.140 | 0.071 | | 0.208 | | 0.010 | | -0.069 | 0.088 | |  |
| Wholesale and Retail Trade Motor Repair (WRTMR) | Pre-pandemic | No | 0.123 | 0.084 | | 0.162 | |  | |  |  | |  |
|  | Pre-pandemic | Yes | 0.131 | 0.085 | | 0.176 | | 0.008 | | -0.048 | 0.064 | |  |
|  | pandemic | No | 0.167 | 0.129 | | 0.205 | |  | |  |  | |  |
|  | pandemic | Yes | 0.137 | 0.097 | | 0.178 | | -0.030 | | -0.084 | 0.025 | |  |
| Transportation and Storage (TS) | Pre-pandemic | No | 0.111 | 0.051 | | 0.172 | |  | |  |  | |  |
|  | Pre-pandemic | Yes | 0.096 | 0.052 | | 0.141 | | -0.015 | | -0.085 | 0.055 | |  |
|  | pandemic | No | 0.104 | 0.056 | | 0.153 | |  | |  |  | |  |
|  | pandemic | Yes | 0.155 | 0.104 | | 0.206 | | 0.051 | | -0.019 | 0.121 | |  |
| Accommodation and Food Services (AF) | Pre-pandemic | No | 0.172 | 0.102 | | 0.242 | |  | |  |  | |  |
|  | Pre-pandemic | Yes | 0.070 | 0.005 | | 0.135 | | -0.102 | | -0.196 | -0.008 | |  |
|  | pandemic | No | 0.177 | 0.117 | | 0.236 | |  | |  |  | |  |
|  | pandemic | Yes | 0.174 | 0.090 | | 0.258 | | -0.003 | | -0.105 | 0.100 | |  |
| Information and Communication (IC) | Pre-pandemic | No | 0.090 | 0.041 | | 0.138 | |  | |  |  | |  |
|  | Pre-pandemic | Yes | 0.233 | 0.078 | | 0.388 | | 0.143 | | -0.016 | 0.303 | |  |
|  | pandemic | No | 0.172 | 0.132 | | 0.211 | |  | |  |  | |  |
|  | pandemic | Yes | 0.144 | 0.086 | | 0.202 | | -0.028 | | -0.098 | 0.041 | |  |
| Financial and Insurance Activities (FI) | Pre-pandemic | No | 0.158 | 0.097 | | 0.220 | |  | |  |  | |  |
|  | Pre-pandemic | Yes | 0.104 | 0.045 | | 0.162 | | -0.055 | | -0.135 | 0.026 | |  |
|  | pandemic | No | 0.122 | 0.087 | | 0.158 | |  | |  |  | |  |
|  | pandemic | Yes | 0.103 | 0.061 | | 0.145 | | -0.019 | | -0.073 | 0.034 | |  |
| Real Estate Activities (RE) | Pre-pandemic | No | 0.100 | 0.014 | | 0.186 | |  | |  |  | |  |
|  | Pre-pandemic | Yes | 0.316 | 0.163 | | 0.468 | | 0.215 | | 0.035 | 0.395 | |  |
|  | pandemic | No | 0.168 | 0.079 | | 0.257 | |  | |  |  | |  |
|  | pandemic | Yes | 0.100 | 0.017 | | 0.183 | | -0.068 | | -0.191 | 0.055 | |  |
| Professional Scientific & Technical (PST) | Pre-pandemic | No | 0.103 | 0.072 | | 0.133 | |  | |  |  | |  |
|  | Pre-pandemic | Yes | 0.106 | 0.052 | | 0.160 | | 0.004 | | -0.055 | 0.062 | |  |
|  | pandemic | No | 0.153 | 0.123 | | 0.184 | |  | |  |  | |  |
|  | pandemic | Yes | 0.165 | 0.126 | | 0.204 | | 0.011 | | -0.037 | 0.060 | |  |
| Administrative and Support Services (AS) | Pre-pandemic | No | 0.075 | 0.034 | | 0.115 | |  | |  |  | |  |
|  | Pre-pandemic | Yes | 0.089 | 0.010 | | 0.169 | | 0.015 | | -0.071 | 0.100 | |  |
|  | pandemic | No | 0.124 | 0.083 | | 0.165 | |  | |  |  | |  |
|  | pandemic | Yes | 0.133 | 0.092 | | 0.174 | | 0.009 | | -0.048 | 0.066 | |  |
| Public Administration and Defence (PAD) | Pre-pandemic | No | 0.058 | -0.002 | | 0.118 | |  | |  |  | |  |
|  | Pre-pandemic | Yes | 0.120 | 0.091 | | 0.150 | | 0.062 | | -0.003 | 0.127 | |  |
|  | pandemic | No | 0.126 | 0.074 | | 0.178 | |  | |  |  | |  |
|  | pandemic | Yes | 0.153 | 0.127 | | 0.179 | | 0.027 | | -0.030 | 0.085 | |  |
| Education (EDU) | Pre-pandemic | No | 0.067 | 0.033 | | 0.102 | |  | |  |  | |  |
|  | Pre-pandemic | Yes | 0.118 | 0.092 | | 0.143 | | 0.051 | | 0.012 | 0.089 | |  |
|  | pandemic | No | 0.162 | 0.122 | | 0.202 | |  | |  |  | |  |
|  | pandemic | Yes | 0.155 | 0.133 | | 0.177 | | -0.007 | | -0.051 | 0.037 | |  |
| Human Health and Social Work Activities (HHSW) | Pre-pandemic | No | 0.119 | 0.086 | | 0.152 | |  | |  |  | |  |
|  | Pre-pandemic | Yes | 0.129 | 0.103 | | 0.155 | | 0.010 | | -0.027 | 0.047 | |  |
|  | pandemic | No | 0.140 | 0.110 | | 0.170 | |  | |  |  | |  |
|  | pandemic | Yes | 0.162 | 0.139 | | 0.185 | | 0.022 | | -0.015 | 0.059 | |  |
| Other Services (OTH) | Pre-pandemic | No | 0.134 | 0.075 | | 0.193 | |  | |  |  | |  |
|  | Pre-pandemic | Yes | 0.105 | 0.047 | | 0.163 | | -0.029 | | -0.110 | 0.052 | |  |
|  | pandemic | No | 0.137 | 0.108 | | 0.165 | |  | |  |  | |  |
|  | pandemic | Yes | 0.124 | 0.091 | | 0.156 | | -0.013 | | -0.055 | 0.029 | |  |
|  |  |  | **Trade union membership**  **(Sample restricted to trade union presence)** | | | | | | | | | | |
|  |  | **Trade Union** |  | | **95 % CI** | | | |  | **95 % CI** | | | |
|  | **Time Period** |  | **MM** | | **Lower** | | **Higher** | | **AME** | **Lower** | | **Higher** | |
| Mining, Energy and Water Supply (MEWS) | Pre-pandemic | No | 0.141 | | -0.032 | | 0.313 | |  |  | |  | |
|  | Pre-pandemic | Yes | 0.197 | | 0.044 | | 0.350 | | 0.056 | -0.174 | | 0.286 | |
|  | pandemic | No | 0.157 | | 0.018 | | 0.295 | |  |  | |  | |
|  | pandemic | Yes | 0.146 | | 0.067 | | 0.226 | | -0.010 | -0.168 | | 0.148 | |
| Manufacturing (MAN) | Pre-pandemic | No | 0.043 | | -0.003 | | 0.088 | |  |  | |  | |
|  | Pre-pandemic | Yes | 0.107 | | 0.045 | | 0.169 | | 0.064 | -0.008 | | 0.137 | |
|  | pandemic | No | 0.099 | | 0.040 | | 0.158 | |  |  | |  | |
|  | pandemic | Yes | 0.116 | | 0.059 | | 0.173 | | 0.017 | -0.064 | | 0.098 | |
| Construction (CON) | Pre-pandemic | No | 0.221 | | 0.042 | | 0.400 | |  |  | |  | |
|  | Pre-pandemic | Yes | 0.156 | | -0.007 | | 0.320 | | -0.065 | -0.305 | | 0.175 | |
|  | pandemic | No | 0.128 | | 0.048 | | 0.209 | |  |  | |  | |
|  | pandemic | Yes | 0.153 | | 0.058 | | 0.248 | | 0.025 | -0.099 | | 0.148 | |
| Wholesale and Retail Trade Motor Repair (WRTMR) | Pre-pandemic | No | 0.156 | | 0.073 | | 0.238 | |  |  | |  | |
|  | Pre-pandemic | Yes | 0.132 | | 0.070 | | 0.194 | | -0.024 | -0.128 | | 0.081 | |
|  | pandemic | No | 0.168 | | 0.108 | | 0.227 | |  |  | |  | |
|  | pandemic | Yes | 0.140 | | 0.085 | | 0.195 | | -0.027 | -0.106 | | 0.052 | |
| Transportation and Storage (TS) | Pre-pandemic | No | 0.064 | | 0.006 | | 0.122 | |  |  | |  | |
|  | Pre-pandemic | Yes | 0.130 | | 0.064 | | 0.196 | | 0.066 | -0.019 | | 0.151 | |
|  | pandemic | No | 0.133 | | 0.051 | | 0.215 | |  |  | |  | |
|  | pandemic | Yes | 0.178 | | 0.115 | | 0.241 | | 0.045 | -0.057 | | 0.148 | |
| Accommodation and Food Services (AF) | Pre-pandemic | No | 0.107 | | 0.001 | | 0.214 | |  |  | |  | |
|  | Pre-pandemic | Yes | 0.008 | | -0.008 | | 0.024 | | -0.100 | -0.208 | | 0.008 | |
|  | pandemic | No | 0.183 | | 0.098 | | 0.268 | |  |  | |  | |
|  | pandemic | Yes | 0.157 | | 0.028 | | 0.287 | | -0.026 | -0.181 | | 0.129 | |
| Information and Communication (IC) | Pre-pandemic | No | 0.175 | | 0.027 | | 0.323 | |  |  | |  | |
|  | Pre-pandemic | Yes | 0.266 | | 0.047 | | 0.486 | | 0.092 | -0.167 | | 0.350 | |
|  | pandemic | No | 0.201 | | 0.108 | | 0.294 | |  |  | |  | |
|  | pandemic | Yes | 0.106 | | 0.048 | | 0.165 | | -0.095 | -0.204 | | 0.015 | |
| Financial and Insurance Activities (FI) | Pre-pandemic | No | 0.062 | | 0.019 | | 0.106 | |  |  | |  | |
|  | Pre-pandemic | Yes | 0.171 | | 0.036 | | 0.306 | | 0.109 | -0.030 | | 0.247 | |
|  | pandemic | No | 0.113 | | 0.063 | | 0.164 | |  |  | |  | |
|  | pandemic | Yes | 0.077 | | 0.023 | | 0.132 | | -0.036 | -0.110 | | 0.038 | |
| Real Estate Activities (RE) | Pre-pandemic | No | 0.277 | | 0.104 | | 0.450 | |  |  | |  | |
|  | Pre-pandemic | Yes | 0.367 | | 0.108 | | 0.625 | | 0.090 | -0.214 | | 0.394 | |
|  | pandemic | No | 0.138 | | 0.013 | | 0.263 | |  |  | |  | |
|  | pandemic | Yes | 0.070 | | 0.018 | | 0.123 | | -0.068 | -0.201 | | 0.066 | |
| Professional Scientific & Technical (PST) | Pre-pandemic | No | 0.114 | | 0.033 | | 0.196 | |  |  | |  | |
|  | Pre-pandemic | Yes | 0.114 | | 0.032 | | 0.196 | | 0.000 | -0.113 | | 0.112 | |
|  | pandemic | No | 0.197 | | 0.137 | | 0.257 | |  |  | |  | |
|  | pandemic | Yes | 0.157 | | 0.109 | | 0.206 | | -0.040 | -0.116 | | 0.036 | |
| Administrative and Support Services (AS) | Pre-pandemic | No | 0.042 | | -0.010 | | 0.094 | |  |  | |  | |
|  | Pre-pandemic | Yes | 0.220 | | -0.009 | | 0.450 | | 0.179 | -0.049 | | 0.407 | |
|  | pandemic | No | 0.146 | | 0.092 | | 0.200 | |  |  | |  | |
|  | pandemic | Yes | 0.104 | | 0.058 | | 0.149 | | -0.042 | -0.112 | | 0.028 | |
| Public Administration and Defence (PAD) | Pre-pandemic | No | 0.116 | | 0.073 | | 0.160 | |  |  | |  | |
|  | Pre-pandemic | Yes | 0.134 | | 0.095 | | 0.173 | | 0.018 | -0.035 | | 0.071 | |
|  | pandemic | No | 0.137 | | 0.103 | | 0.172 | |  |  | |  | |
|  | pandemic | Yes | 0.170 | | 0.134 | | 0.207 | | 0.033 | -0.016 | | 0.081 | |
| Education (EDU) | Pre-pandemic | No | 0.125 | | 0.085 | | 0.166 | |  |  | |  | |
|  | Pre-pandemic | Yes | 0.125 | | 0.091 | | 0.159 | | 0.000 | -0.046 | | 0.046 | |
|  | pandemic | No | 0.159 | | 0.126 | | 0.192 | |  |  | |  | |
|  | pandemic | Yes | 0.163 | | 0.136 | | 0.190 | | 0.004 | -0.037 | | 0.045 | |
| Human Health and Social Work Activities (HHSW) | Pre-pandemic | No | 0.143 | | 0.100 | | 0.186 | |  |  | |  | |
|  | Pre-pandemic | Yes | 0.129 | | 0.096 | | 0.163 | | -0.013 | -0.061 | | 0.035 | |
|  | pandemic | No | 0.169 | | 0.137 | | 0.202 | |  |  | |  | |
|  | pandemic | Yes | 0.162 | | 0.135 | | 0.190 | | -0.007 | -0.048 | | 0.034 | |
| Other Services (OTH) | Pre-pandemic | No | 0.044 | | 0.010 | | 0.078 | |  |  | |  | |
|  | Pre-pandemic | Yes | 0.241 | | 0.107 | | 0.375 | | 0.197 | 0.060 | | 0.333 | |
|  | pandemic | No | 0.112 | | 0.067 | | 0.157 | |  |  | |  | |
|  | pandemic | Yes | 0.144 | | 0.102 | | 0.186 | | 0.032 | -0.029 | | 0.093 | |

Note: All models are adjusted for gender (ref.: female), age, age square, ethnicity (ref.: white), industrial group (ref.: hotel and accommodation), UK country of residence (ref.: England), education (ref.: university degree), financial situation (ref.: doing all right), baseline (pre-pandemic) health condition (ref.: longstanding illness or impairment) and size of workplace (ref.:1 to 34 employees). Secular effects across time are accounted for by the inclusion of dummy variables for each pre-pandemic Wave and COVID-19 Sweep

# Supplementary file S3. Sample characteristics for trade union presence and membership

## Table S3.1. Sample characteristics for union presence in the pre and pandemic USoc Waves*

|  |  | **Trade Union presence**** | | | **Trade Union membership**** | | |
| --- | --- | --- | --- | --- | --- | --- | --- |
|  |  | **Pre-Pandemic** | **Pandemic** | **Total** | **Pre-Pandemic** | **Pandemic** | **Total** |
| **Outcome** | *GHQ case* |  |  |  |  |  |  |
|  | No | 11,636 | 27,254 | 38,890 | 6,078 | 15,510 | 21,588 |
|  | % | 82.3 (81.6) | 76.2 (74.6) | 77.9 (76.5) | 81.4 (80.6) | 75.6 (74.7) | 77.2 (76.2) |
|  | Yes | 2,506 | 8,519 | 11,025 | 1,389 | 4,994 | 6,383 |
|  | % | 17.7 (18.4) | 23.8 (25.4) | 22.1 (23.5) | 18.6 (19.4) | 24.4 (25.3) | 22.8 (23.8) |
|  | **Total** | **14,142** | **35,773** | **49,915** | **7,467** | **20,504** | **27,971** |
|  | **%** | **100.0** | **100.0** | **100.0** | **100.0** | **100.0** | **100.0** |
| **Confounders** | *Sex* |  |  |  |  |  |  |
|  | Male | 5,815 | 14,225 | 20,040 | 2,672 | 7,081 | 9,753 |
|  | % | 41.1 (44.1) | 39.8 (46.3) | 40.1 (45.7) | 35.8 (38.9) | 34.5 (41.5) | 34.9 (40.8) |
|  | Female | 8,327 | 21,548 | 29,875 | 4,795 | 13,423 | 18,218 |
|  | % | 58.9 (55.9) | 60.2 (53.7) | 59.9 (54.3) | 64.2 (61.1) | 65.5 (58.5) | 65.1 (59.2) |
|  | *Race* |  |  |  |  |  |  |
|  | White | 13,040 | 33,063 | 46,103 | 6,865 | 18,924 | 25,789 |
|  | % | 92.2 (94.6) | 92.4 (94.4) | 92.4 (94.5) | 91.9 (94.8) | 92.3 (94.7) | 92.2 (94.7) |
|  | Non-white | 1,102 | 2,710 | 3,812 | 602 | 1,580 | 2,182 |
|  | % | 7.8 (5.4) | 7.6 (5.6) | 7.6 (5.5) | 8.1 (5.2) | 7.7 (5.3) | 7.8 (5.3) |
|  | *UK country* |  |  |  |  |  |  |
|  | England | 11,238 | 28,932 | 40,170 | 5,715 | 16,143 | 21,858 |
|  | % | 79.5 (85.7) | 80.9 (85.2) | 80.5 (85.3) | 76.5 (83.4) | 78.7 (83.7) | 78.1 (83.6) |
|  | Wales | 926 | 2,165 | 3,091 | 571 | 1,408 | 1,979 |
|  | % | 6.5 (4.5) | 6.1 (4.7) | 6.2 (4.7) | 7.6 (5.3) | 6.9 (5.1) | 7.1 (5.2) |
|  | Scotland | 1,278 | 3,204 | 4,482 | 763 | 2,007 | 2,770 |
|  | % | 9.0 (7.8) | 9.0 (7.7) | 9.0 (7.7) | 10.2 (9.1) | 9.8 (8.9) | 9.9 (8.9) |
|  | Northern Ireland | 700 | 1,472 | 2,172 | 418 | 946 | 1,364 |
|  | % | 4.9 (2.0) | 4.1 (2.4) | 4.4 (2.3) | 5.6 (2.2) | 4.6 (2.3) | 4.9 (2.3) |
|  | *Qualifications* |  |  |  |  |  |  |
|  | Degree | 8,110 | 20,756 | 28,866 | 4,562 | 12,540 | 17,102 |
|  | % | 57.3 (56.4) | 58.0 (51.6) | 57.8 (52.9) | 61.1 (59.7) | 61.2 (55.1) | 61.1 (56.3) |
|  | A-Level | 2,904 | 7,376 | 10,280 | 1,470 | 4,043 | 5,513 |
|  | % | 20.5 (20.8) | 20.6 (23.0) | 20.6 (22.5) | 19.7 (19.8) | 19.7 (21.1) | 19.7 (20.8) |
|  | GCSE | 2,347 | 5,848 | 8,195 | 1,095 | 3,046 | 4,141 |
|  | % | 16.6 (17.0) | 16.3 (19.5) | 16.4 (18.8) | 14.7 (15.6) | 14.9 (18.8) | 14.8 (18.0) |
|  | Other | 596 | 1,403 | 1,999 | 259 | 668 | 927 |
|  | % | 4.2 (4.5) | 3.9 (4.6) | 4.0 (4.6) | 3.5 (3.8) | 3.3 (3.8) | 3.3 (3.8) |
|  | None | 185 | 390 | 575 | 81 | 207 | 288 |
|  | % | 1.3 (1.3) | 1.1 (1.2) | 1.2 (1.2) | 1.1 (1.2) | 1.0 (1.2) | 1.0 (1.2) |
|  | *Financial situation* |  |  |  |  |  |  |
|  | Good | 10,652 | 28,892 | 39,544 | 5,580 | 16,788 | 22,368 |
|  | % | 75.3 (74.4) | 80.8 (77.3) | 79.2 (76.5) | 74.7 (73.5) | 81.9 (78.6) | 80.0 (77.3) |
|  | Bad | 3,490 | 6,881 | 10,371 | 1,887 | 3,716 | 5,603 |
|  | % | 24.7 (25.6) | 19.2 (22.7) | 20.8 (23.5) | 25.3 (26.5) | 18.1 (21.4) | 20.0 (22.7) |
|  | *Past health condition* |  |  |  |  |  |  |
|  | No | 10,325 | 25,776 | 36,101 | 5,377 | 14,537 | 19,914 |
|  | % | 73.0 (72.6) | 72.1 (71.6) | 72.3 (71.9) | 72.0 (71.4) | 70.9 (70.9) | 71.2 (71.0) |
|  | Yes | 3,817 | 9,997 | 13,814 | 2,090 | 5,967 | 8,057 |
|  | % | 27.0 (27.4) | 27.9 (28.4) | 27.7 (28.2) | 28.0 (28.6) | 29.1 (29.1) | 28.8 (29.0) |
|  | *Job size* |  |  |  |  |  |  |
|  | 1 to 24 employees | 3,701 | 9,197 | 12,898 | 1,059 | 3,248 | 4,307 |
|  | % | 26.2 (26.6) | 25.7 (27.4) | 25.8 (27.2) | 26.2 (14.5) | 25.7 (15.9) | 25.8 (15.6) |
|  | 25 to 199 employees | 5,301 | 12,724 | 18,025 | 2,855 | 7,358 | 10,213 |
|  | % | 37.5 (37.8) | 35.6 (36.3) | 36.1 (36.7) | 37.5 (38.0) | 35.6 (36.9) | 36.1 (37.2) |
|  | More than 200 employees | 5,140 | 13,852 | 18,992 | 3,553 | 9,898 | 13,451 |
|  | % | 36.4 (35.6) | 38.7 (36.4) | 38.1 (36.2) | 36.4 (47.5) | 38.7 (47.2) | 38.1 (47.3) |
|  | **Total** | **14,142** | **35,773** | **49,915** | **7,467** | **20,504** | **27,971** |
|  | **%** | **100.0** | **100.0** | **100.0** | **100.0** | **100.0** | **100.0** |
|  | *Age (continuous)* | **Mean**  **sd** | **Mean**  **sd** | **Mean**  **sd** | **Mean**  **sd** | **Mean**  **sd** | **Mean**  **sd** |
|  |  | 45.7 (44.59)  11.3 (11.8) | 47.7 (44.9)  11.3 (12.3) | 47.2 (44.8)  11.4 (12.2) | 46.1 (45.2)  10.6 (10.8) | 48.2 (45.9)  10.8 (11.7) | 47.7 (45.8)  10.8 (11.6) |

*Calculation using the weighted Sample (in brackets))

## Table S3.2. Observations for Industry (SIC-2007) for union presence in pre-pandemic and pandemic Waves (Unweighted and Weighted Sample (in brackets))

| **Industry SIC-2007** | **Trade Union presence** | | | **Trade Union membership** | | |
| --- | --- | --- | --- | --- | --- | --- |
|  | Pre-pandemic | Pandemic | Total | Pre-pandemic | Pandemic | Total |
| Mining, Energy and Water Supply | 210 | 727 | 937 | 118 | 348 | 466 |
| % Unweighted (Weighted) | 1.5(1.4) | 2.0(2.2) | 1.9(1.9) | 1.58(1.4) | 1.73(1.9) | 1.68(1.8) |
| Manufacturing | 1,319 | 2,722 | 4,041 | 381 | 866 | 1,247 |
| % Unweighted (Weighted) | 9.3(9.3) | 7.6(8.0) | 8.1(8.4) | 5.16(5.0) | 4.17(4.8) | 4.43(4.9) |
| Construction | 456 | 1,252 | 1,734 | 99 | 304 | 403 |
| % Unweighted (Weighted) | 3.2(3.4) | 3.5(4.6) | 3.4(4.3) | 1.3(1.4) | 1.5(2.0) | 1.5(1.8) |
| Wholesale and Retail Trade Motor Repair | 1,599 | 3,680 | 5,279 | 532 | 1,397 | 1,929 |
| % Unweighted (Weighted) | 11.3(12.4) | 10.3(12.6) | 10.6(12.6) | 7.2(8.7) | 6.9(9.2) | 7.0(9.0) |
| Transportation and Storage | 574 | 1,224 | 1,798 | 340 | 775 | 1,115 |
| % Unweighted (Weighted) | 4.1(4.1) | 3.4(4.0) | 3.6(4.0) | 4.6(4.6) | 3.8(4.7) | 4.0(4.7) |
| Accommodation and Food Services | 393 | 800 | 1,193 | 78 | 198 | 276 |
| % Unweighted (Weighted) | 2.8(3.0) | 2.2(2.7) | 2.4(2.8) | 1.1(1.1) | 1.0(1.1) | 1.0(1.1) |
| Information and Communication | 574 | 1,616 | 2,190 | 154 | 575 | 729 |
| % Unweighted (Weighted) | 4.1(4.1) | 4.5(4.5) | 4.4(4.4) | 2.1(2.4) | 2.8(3.1) | 2.6(2.9) |
| Financial and Insurance Activities | 537 | 1,749 | 2,286 | 224 | 774 | 998 |
| % Unweighted (Weighted) | 3.8(4.1) | 4.9(4.5) | 4.6(4.4) | 3.0(3.1) | 3.8(3.5) | 3.6(3.4) |
| Real Estate Activities | 164 | 329 | 493 | 66 | 102 | 168 |
| % Unweighted (Weighted) | 1.2(1.2) | 0.9(0.8) | 1.0(0.9) | 0.9(1.0) | 0.5(0.5) | 0.6(0.6) |
| Professional Scientific and Technical | 970 | 2,260 | 3,230 | 197 | 830 | 1,027 |
| % Unweighted (Weighted) | 6.9(6.9) | 6.3(5.4) | 6.5(5.8) | 2.6(2.5) | 4.1(3.7) | 3.7(3.4) |
| Administrative and Support Services | 445 | 1,500 | 1,945 | 126 | 887 | 1,013 |
| % Unweighted (Weighted) | 3.2(3.5) | 4.2(4.0) | 3.9(3.9) | 1.7(1.9) | 4.3(4.5) | 3.6(3.8) |
| Public Administration and Defence | 1,426 | 2,575 | 4,001 | 1,282 | 2,326 | 3,608 |
| % Unweighted (Weighted) | 10.1(9.2) | 7.2(6.5) | 8.0(7.2) | 17.0(16.1) | 11.2(10.3) | 12.8(11.8) |
| Education | 2,250 | 6,024 | 8,274 | 1,877 | 5,136 | 7,013 |
| % Unweighted (Weighted) | 15.9(15.5) | 16.8(15.1) | 16.6(15.2) | 25.2(25.1) | 25.1(23.4) | 25.2(23.8) |
| Human Health and Social Work Activities | 2,717 | 6,253 | 8,970 | 1,803 | 4,567 | 6,370 |
| % Unweighted (Weighted) | 19.2(18.1) | 17.5(15.9) | 18.0(16.5) | 24.2(23.0) | 22.1(19.5) | 22.7(20.4) |
| Other Services | 508 | 3,062 | 3,570 | 190 | 1,419 | 1,609 |
| % Unweighted (Weighted) | 3.6(3.8) | 8.6(9.5) | 7.2(7.9) | 2.5(2.8) | 7.1(7.9) | 5.8(6.6) |
| **Total** | **14,142** | **35,773** | **49,915** | **7,467** | **20,504** | **27,971** |
| **%** | **100.00** | **100.00** | **100.00** | **100.00** | **100.00** | **100.00** |

# Supplementary file S4. Sensitivity analyses

## S4.1. Union presence – adjusted model (2-way interaction), excluding union members

|  |  | **Adjusted model (weighted)** | | | | | |
| --- | --- | --- | --- | --- | --- | --- | --- |
|  |  | **OR** | **std. err.** | **z** | **P>z** | **[95% conf.** | **interval]** |
| sex |  | 2.49 | 0.20 | 11.23 | 0.00 | 2.12 | 2.92 |
| age |  | 1.02 | 0.02 | 0.84 | 0.40 | 0.98 | 1.06 |
| age_square | | 1.00 | 0.00 | -2.22 | 0.03 | 1.00 | 1.00 |
| Ethnicity | | 0.88 | 0.13 | -0.87 | 0.38 | 0.66 | 1.17 |
| **During pandemic: 1** | | **1.53** | **0.20** | **3.30** | **0.00** | **1.19** | **1.98** |
| **Union presence: yes** | | **0.97** | **0.11** | **-0.30** | **0.76** | **0.77** | **1.21** |
| **During pandemic: 1 # Union presence: yes** | | **0.94** | **0.12** | **-0.47** | **0.64** | **0.74** | **1.20** |
| Industry: | |  |  |  |  |  |  |
|  | Mining, Energy and Water Supply | 1.18 | 0.35 | 0.55 | 0.58 | 0.66 | 2.10 |
|  | Manufacturing | 0.65 | 0.14 | -2.01 | 0.04 | 0.43 | 0.99 |
|  | Construction | 0.63 | 0.15 | -1.91 | 0.06 | 0.39 | 1.01 |
|  | Wholesale and Retail Trade Motor Repair | 0.84 | 0.17 | -0.83 | 0.40 | 0.57 | 1.26 |
|  | Transportation and Storage | 0.53 | 0.14 | -2.37 | 0.02 | 0.31 | 0.89 |
|  | Information and Communication | 0.85 | 0.19 | -0.72 | 0.47 | 0.55 | 1.32 |
|  | Financial and Insurance Activities | 0.64 | 0.16 | -1.82 | 0.07 | 0.40 | 1.03 |
|  | Real Estate Activities | 0.98 | 0.30 | -0.05 | 0.96 | 0.54 | 1.79 |
|  | Professional Scientific and Technical | 0.76 | 0.16 | -1.34 | 0.18 | 0.51 | 1.13 |
|  | Administrative and Support Services | 0.59 | 0.14 | -2.30 | 0.02 | 0.38 | 0.93 |
|  | Public Administration and Defence | 0.65 | 0.15 | -1.92 | 0.06 | 0.42 | 1.01 |
|  | Education | 0.74 | 0.15 | -1.50 | 0.13 | 0.49 | 1.10 |
|  | Human Health and Social Work Activities | 0.75 | 0.15 | -1.43 | 0.15 | 0.51 | 1.11 |
|  | Other Services | 0.62 | 0.13 | -2.32 | 0.02 | 0.41 | 0.93 |
| Country: | |  |  |  |  |  |  |
|  | Wales | 1.43 | 0.23 | 2.21 | 0.03 | 1.04 | 1.97 |
|  | Scotland | 1.04 | 0.15 | 0.28 | 0.78 | 0.78 | 1.39 |
|  | Northern Ireland | 0.91 | 0.18 | -0.50 | 0.61 | 0.62 | 1.33 |
| Highest level of education: | |  |  |  |  |  |  |
|  | A-Level | 0.70 | 0.07 | -3.82 | 0.00 | 0.58 | 0.84 |
|  | GCSE | 0.82 | 0.09 | -1.85 | 0.06 | 0.67 | 1.01 |
|  | Other | 0.60 | 0.12 | -2.45 | 0.01 | 0.40 | 0.90 |
|  | None | 0.35 | 0.12 | -2.98 | 0.00 | 0.17 | 0.70 |
| Self-reported financial conditions: bad | | 2.27 | 0.19 | 9.69 | 0.00 | 1.92 | 2.68 |
| Health condition: yes | | 2.16 | 0.18 | 9.44 | 0.00 | 1.84 | 2.53 |
| Company size: | |  |  |  |  |  |  |
|  | 25-199 | 0.87 | 0.08 | -1.46 | 0.14 | 0.72 | 1.05 |
|  | More than 200 | 0.96 | 0.09 | -0.47 | 0.64 | 0.79 | 1.15 |
| Wave Number | |  |  |  |  |  |  |
|  | Wave 10 | 0.92 | 0.09 | -0.80 | 0.42 | 0.76 | 1.12 |
|  | Wave 11 | 1.31 | 0.14 | 2.53 | 0.01 | 1.06 | 1.63 |
|  | COVID-19 Sweeps |  |  |  |  |  |  |
|  | Apr-20 | 2.23 | 0.25 | 7.14 | 0.00 | 1.79 | 2.78 |
|  | May-20 | 1.83 | 0.21 | 5.22 | 0.00 | 1.46 | 2.29 |
|  | Jun-20 | 1.69 | 0.20 | 4.31 | 0.00 | 1.33 | 2.14 |
|  | Jul-20 | 1.01 | 0.12 | 0.10 | 0.92 | 0.81 | 1.26 |
|  | Sep-20 | 0.96 | 0.12 | -0.36 | 0.72 | 0.75 | 1.22 |
|  | Nov-20 | 1.89 | 0.24 | 5.00 | 0.00 | 1.47 | 2.43 |
|  | Jan-21 | 1.63 | 0.20 | 4.07 | 0.00 | 1.29 | 2.07 |
|  | Mar-21 | 1.27 | 0.15 | 1.98 | 0.05 | 1.00 | 1.61 |
|  | Sep-21 | 1.00 | (omitted) |  |  |  |  |
| Constant |  | 0.03 | 0.02 | -6.77 | 0.00 | 0.01 | 0.08 |

Note: Observations=39,190; Individuals=4,405 (Minimum=1; Average= 7.8; Maximum=12). **Exposure is in bold letters.** Reference categories: gender (ref.: female), age, age square, ethnicity (ref.: white), Interaction : ref (pre-pandemic-No presence of trade union), industrial group (ref.: hotel and accommodation), UK country of residence (ref.: England), education (ref.: university degree), financial situation (ref.: Good financial situation), health condition (ref.: Not having longstanding illness or impairment) and size of workplace (ref.:1 to 34 employees)

## S4.2. Union presence – adjusted model (3-way interaction), excluding union members

|  |  | **Adjusted model (weighted) -- three-ways interaction** | | | | | |
| --- | --- | --- | --- | --- | --- | --- | --- |
|  |  | **OR** | **std. err.** | **z** | **P>z** | **[95% conf.** | **interval]** |
| Sex |  | 2.48 | 0.20 | 11.13 | 0.00 | 2.11 | 2.91 |
| Age | | 1.02 | 0.02 | 0.94 | 0.35 | 0.98 | 1.06 |
| Age square | | 1.00 | 0.00 | -2.33 | 0.02 | 1.00 | 1.00 |
| Ethnicity | | 0.87 | 0.13 | -0.98 | 0.33 | 0.65 | 1.15 |
| **During pandemic: 1 # Industry:** | |  |  |  |  |  |  |
|  | **Pre-Pandemic-#Mining, Energy and Water Supply** | 3.04 | 1.73 | 1.95 | 0.05 | 0.99 | 9.29 |
|  | **Post-Pandemic-#Mining, Energy and Water Supply** | 2.31 | 0.74 | 2.62 | 0.01 | 1.23 | 4.34 |
|  | **Pre-Pandemic-#Manufacturing** | 0.85 | 0.21 | -0.67 | 0.51 | 0.52 | 1.38 |
|  | **Post-Pandemic-#Manufacturing** | 1.46 | 0.37 | 1.51 | 0.13 | 0.89 | 2.40 |
|  | **Pre-Pandemic-#Construction** | 0.72 | 0.23 | -1.03 | 0.31 | 0.38 | 1.35 |
|  | **Post-Pandemic-#Construction** | 1.25 | 0.34 | 0.82 | 0.41 | 0.73 | 2.15 |
|  | **Pre-Pandemic-#Wholesale and Retail Trade Motor Repair** | 1.11 | 0.28 | 0.40 | 0.69 | 0.67 | 1.83 |
|  | **Post-Pandemic-#Wholesale and Retail Trade Motor Repair** | 1.61 | 0.42 | 1.81 | 0.07 | 0.96 | 2.68 |
|  | **Pre-Pandemic-#Transportation and Storage** | 1.00 | 0.38 | 0.01 | 0.99 | 0.48 | 2.10 |
|  | **Post-Pandemic-#Transportation and Storage** | 0.91 | 0.32 | -0.27 | 0.79 | 0.46 | 1.81 |
|  | **Pre-Pandemic-#Accommodation and Food Services** | 1.61 | 0.50 | 1.51 | 0.13 | 0.87 | 2.97 |
|  | **Post-Pandemic-#Accommodation and Food Services** | 1.81 | 0.56 | 1.94 | 0.05 | 0.99 | 3.31 |
|  | **Pre-Pandemic-#Information and Communication** | 0.75 | 0.27 | -0.79 | 0.43 | 0.37 | 1.52 |
|  | **Post-Pandemic-#Information and Communication** | 1.74 | 0.42 | 2.27 | 0.02 | 1.08 | 2.80 |
|  | **Pre-Pandemic-#Financial and Insurance Activities** | 1.49 | 0.46 | 1.31 | 0.19 | 0.82 | 2.73 |
|  | **Post-Pandemic-#Financial and Insurance Activities** | 1.11 | 0.30 | 0.37 | 0.71 | 0.65 | 1.88 |
|  | **Pre-Pandemic-#Real Estate Activities** | 1.07 | 0.47 | 0.16 | 0.87 | 0.45 | 2.55 |
|  | **Post-Pandemic-#Real Estate Activities** | 1.78 | 0.72 | 1.41 | 0.16 | 0.80 | 3.95 |
|  | **Pre-Pandemic-#Professional Scientific and Technical** | 0.87 | 0.20 | -0.62 | 0.54 | 0.55 | 1.37 |
|  | **Post-Pandemic-#Professional Scientific and Technical** | 1.47 | 0.34 | 1.66 | 0.10 | 0.93 | 2.32 |
|  | **Pre-Pandemic-#Administrative and Support Services** | 0.59 | 0.21 | -1.47 | 0.14 | 0.30 | 1.19 |
|  | **Post-Pandemic-#Administrative and Support Services** | 1.14 | 0.31 | 0.47 | 0.64 | 0.66 | 1.96 |
|  | **Pre-Pandemic-#Public Administration and Defence** | 0.44 | 0.27 | -1.35 | 0.18 | 0.13 | 1.46 |
|  | **Post-Pandemic-#Public Administration and Defence** | 1.22 | 0.39 | 0.61 | 0.54 | 0.65 | 2.29 |
|  | **Pre-Pandemic-#Education** | 0.46 | 0.16 | -2.29 | 0.02 | 0.24 | 0.90 |
|  | **Post-Pandemic-#Education** | 1.67 | 0.44 | 1.95 | 0.05 | 1.00 | 2.79 |
|  | **Post-Pandemic-#Human Health and Social Work Activities** | 1.33 | 0.29 | 1.31 | 0.19 | 0.87 | 2.04 |
|  | **Pre-Pandemic-#Other Services** | 1.17 | 0.38 | 0.49 | 0.63 | 0.62 | 2.20 |
|  | **Post-Pandemic-#Other Services** | 1.30 | 0.29 | 1.17 | 0.24 | 0.84 | 2.02 |
| **Union presence: yes** | | 1.13 | 0.27 | 0.50 | 0.62 | 0.70 | 1.81 |
| **During pandemic: 1 # Industry # Union presence** | |  |  |  |  |  |  |
|  | **Pre-Pandemic-#Mining, Energy and Water Supply#Yes** | 0.35 | 0.35 | -1.06 | 0.29 | 0.05 | 2.43 |
|  | **Post-Pandemic-#Mining, Energy and Water Supply#Yes** | 0.53 | 0.40 | -0.84 | 0.40 | 0.12 | 2.34 |
|  | **Pre-Pandemic-#Manufacturing#Yes** | 0.31 | 0.19 | -1.90 | 0.06 | 0.09 | 1.04 |
|  | **Post-Pandemic-#Manufacturing#Yes** | 0.34 | 0.18 | -2.08 | 0.04 | 0.13 | 0.94 |
|  | **Pre-Pandemic-#Construction#Yes** | 1.88 | 1.34 | 0.89 | 0.37 | 0.47 | 7.57 |
|  | **Post-Pandemic-#Construction#Yes** | 0.50 | 0.27 | -1.28 | 0.20 | 0.18 | 1.44 |
|  | **Pre-Pandemic-#Wholesale and Retail Trade Motor Repair#Yes** | 1.00 | 0.44 | -0.01 | 0.99 | 0.42 | 2.39 |
|  | **Post-Pandemic-#Wholesale and Retail Trade Motor Repair#Yes** | 0.82 | 0.32 | -0.50 | 0.62 | 0.38 | 1.78 |
|  | **Pre-Pandemic-#Transportation and Storage#Yes** | 0.36 | 0.22 | -1.70 | 0.09 | 0.11 | 1.17 |
|  | **Post-Pandemic-#Transportation and Storage#Yes** | 0.95 | 0.55 | -0.09 | 0.93 | 0.31 | 2.93 |
|  | **Pre-Pandemic-#Accommodation and Food Services#Yes** | 0.45 | 0.31 | -1.16 | 0.25 | 0.12 | 1.72 |
|  | **Post-Pandemic-#Accommodation and Food Services#Yes** | 0.80 | 0.40 | -0.45 | 0.66 | 0.30 | 2.13 |
|  | **Pre-Pandemic-#Information and Communication#Yes** | 1.84 | 1.24 | 0.90 | 0.37 | 0.49 | 6.92 |
|  | **Post-Pandemic-#Information and Communication#Yes** | 0.94 | 0.43 | -0.14 | 0.89 | 0.38 | 2.30 |
|  | **Pre-Pandemic-#Financial and Insurance Activities#Yes** | 0.33 | 0.16 | -2.22 | 0.03 | 0.13 | 0.88 |
|  | **Post-Pandemic-#Financial and Insurance Activities#Yes** | 0.89 | 0.37 | -0.29 | 0.77 | 0.39 | 2.00 |
|  | **Pre-Pandemic-#Real Estate Activities#Yes** | 2.75 | 2.02 | 1.38 | 0.17 | 0.65 | 11.61 |
|  | **Post-Pandemic-#Real Estate Activities#Yes** | 0.54 | 0.47 | -0.71 | 0.48 | 0.10 | 2.94 |
|  | **Pre-Pandemic-#Professional Scientific and Technical#Yes** | 0.83 | 0.43 | -0.35 | 0.72 | 0.31 | 2.27 |
|  | **Post-Pandemic-#Professional Scientific and Technical#Yes** | 1.03 | 0.36 | 0.08 | 0.94 | 0.52 | 2.05 |
|  | **Pre-Pandemic-#Administrative and Support Services#Yes** | 0.40 | 0.32 | -1.16 | 0.25 | 0.08 | 1.88 |
|  | **Post-Pandemic-#Administrative and Support Services#Yes** | 1.04 | 0.40 | 0.09 | 0.93 | 0.48 | 2.22 |
|  | **Pre-Pandemic-#Public Administration and Defence#Yes** | 1.83 | 1.24 | 0.89 | 0.37 | 0.48 | 6.88 |
|  | **Post-Pandemic-#Public Administration and Defence#Yes** | 0.88 | 0.34 | -0.31 | 0.75 | 0.41 | 1.90 |
|  | **Pre-Pandemic-#Education#Yes** | 1.85 | 0.77 | 1.48 | 0.14 | 0.82 | 4.17 |
|  | **Post-Pandemic-#Education#Yes** | 0.73 | 0.24 | -0.94 | 0.35 | 0.39 | 1.40 |
|  | **Post-Pandemic-#Human Health and Social Work Activities#Yes** | 0.98 | 0.28 | -0.07 | 0.94 | 0.56 | 1.73 |
|  | **Pre-Pandemic-#Other Services #Yes** | 0.20 | 0.11 | -2.87 | 0.00 | 0.07 | 0.60 |
|  | **Post-Pandemic-#Other Services #Yes** | 0.58 | 0.22 | -1.44 | 0.15 | 0.28 | 1.21 |
| Country: | |  |  |  |  |  |  |
|  | Wales | 1.44 | 0.24 | 2.21 | 0.03 | 1.04 | 1.98 |
|  | Scotland | 1.03 | 0.15 | 0.17 | 0.87 | 0.76 | 1.38 |
|  | Northern Ireland | 0.91 | 0.18 | -0.48 | 0.63 | 0.62 | 1.34 |
| Highest level of education: | |  |  |  |  |  |  |
|  | A-Level | 0.69 | 0.07 | -3.85 | 0.00 | 0.57 | 0.84 |
|  | GCSE | 0.82 | 0.09 | -1.86 | 0.06 | 0.66 | 1.01 |
|  | Other | 0.61 | 0.13 | -2.37 | 0.02 | 0.41 | 0.92 |
|  | None | 0.36 | 0.13 | -2.86 | 0.00 | 0.18 | 0.72 |
| Self-reported financial conditions: bad | | 2.28 | 0.19 | 9.66 | 0.00 | 1.93 | 2.69 |
| Health condition: yes | | 2.14 | 0.18 | 9.34 | 0.00 | 1.83 | 2.52 |
| Company size: | |  |  |  |  |  |  |
|  | 25-199 | 0.88 | 0.08 | -1.37 | 0.17 | 0.73 | 1.06 |
|  | More than 200 | 0.96 | 0.09 | -0.44 | 0.66 | 0.79 | 1.16 |
| Wave Number | |  |  |  |  |  |  |
|  | Wave 10 | 0.92 | 0.09 | -0.83 | 0.41 | 0.76 | 1.12 |
|  | Wave 11 | 1.33 | 0.14 | 2.68 | 0.01 | 1.08 | 1.65 |
|  | COVID-19 Sweeps |  |  |  |  |  |  |
|  | Apr-20 | 2.25 | 0.25 | 7.21 | 0.00 | 1.80 | 2.80 |
|  | May-20 | 1.84 | 0.21 | 5.29 | 0.00 | 1.47 | 2.31 |
|  | Jun-20 | 1.69 | 0.21 | 4.30 | 0.00 | 1.33 | 2.14 |
|  | Jul-20 | 1.01 | 0.12 | 0.09 | 0.93 | 0.81 | 1.26 |
|  | Sep-20 | 0.95 | 0.12 | -0.38 | 0.70 | 0.74 | 1.22 |
|  | Nov-20 | 1.89 | 0.24 | 4.97 | 0.00 | 1.47 | 2.42 |
|  | Jan-21 | 1.63 | 0.20 | 4.03 | 0.00 | 1.28 | 2.06 |
|  | Mar-21 | 1.27 | 0.16 | 1.97 | 0.05 | 1.00 | 1.62 |
|  | Sep-21 | 1.00 | (omitted) |  |  |  |  |
| Constant | | 0.02 | 0.01 | -7.07 | 0.00 | 0.01 | 0.07 |

Note: Observations=39,190; Individuals=4,405 (Minimum=1; Average= 7.8; Maximum=12. **Exposure is in bold letters**. Reference categories: gender (ref.: female), age, age square, ethnicity (ref.: white), Interaction : ref (pre-pandemic-No presence of trade union-Hotel and Accommodation Industry ), UK country of residence (ref.: England), education (ref.: university degree), financial situation (ref.: Good financial situation), health condition (ref.: Not having longstanding illness or impairment) and size of workplace (ref.:1 to 34 employees),‘#’ indicates interaction effects.

## S4.3. Union presence – adjusted model (2-way interaction), excluding public workplaces

|  |  | **Adjusted model (weighted)** | | | | | |
| --- | --- | --- | --- | --- | --- | --- | --- |
|  |  | **OR** | **std. err.** | **z** | **P>z** | **[95% conf.** | **interval]** |
| sex |  | 2.29 | 0.20 | 9.73 | 0.00 | 1.94 | 2.71 |
| age |  | 1.02 | 0.02 | 0.78 | 0.44 | 0.97 | 1.07 |
| age_square | | 1.00 | 0.00 | -1.96 | 0.05 | 1.00 | 1.00 |
| Ethnicity | | 0.93 | 0.15 | -0.43 | 0.67 | 0.67 | 1.29 |
| **During pandemic: 1** | | **1.55** | **0.22** | **3.13** | **0.00** | **1.18** | **2.03** |
| **Union presence: yes** | | **1.09** | **0.13** | **0.72** | **0.47** | **0.87** | **1.36** |
| **During pandemic: 1 # Union presence: yes** | | **0.85** | **0.11** | **-1.25** | **0.21** | **0.65** | **1.10** |
| Industry: | |  |  |  |  |  |  |
|  | Mining, Energy and Water Supply | 1.36 | 0.36 | 1.16 | 0.25 | 0.81 | 2.30 |
|  | Manufacturing | 0.76 | 0.16 | -1.28 | 0.20 | 0.49 | 1.16 |
|  | Construction | 0.76 | 0.19 | -1.09 | 0.27 | 0.46 | 1.24 |
|  | Wholesale and Retail Trade Motor Repair | 0.99 | 0.21 | -0.07 | 0.94 | 0.65 | 1.49 |
|  | Transportation and Storage | 0.83 | 0.20 | -0.75 | 0.46 | 0.52 | 1.34 |
|  | Information and Communication | 0.89 | 0.21 | -0.52 | 0.60 | 0.56 | 1.40 |
|  | Financial and Insurance Activities | 0.72 | 0.18 | -1.35 | 0.18 | 0.44 | 1.16 |
|  | Real Estate Activities | 1.29 | 0.38 | 0.85 | 0.39 | 0.72 | 2.32 |
|  | Professional Scientific and Technical | 0.88 | 0.19 | -0.59 | 0.55 | 0.58 | 1.34 |
|  | Administrative and Support Services | 0.73 | 0.19 | -1.23 | 0.22 | 0.44 | 1.21 |
|  | Public Administration and Defence | 1.16 | 0.38 | 0.44 | 0.66 | 0.61 | 2.21 |
|  | Education | 0.91 | 0.20 | -0.40 | 0.69 | 0.59 | 1.42 |
|  | Human Health and Social Work Activities | 0.88 | 0.19 | -0.60 | 0.55 | 0.57 | 1.35 |
|  | Other Services | 0.75 | 0.16 | -1.30 | 0.19 | 0.49 | 1.15 |
| Country: | |  |  |  |  |  |  |
|  | Wales | 1.30 | 0.23 | 1.49 | 0.14 | 0.92 | 1.83 |
|  | Scotland | 0.90 | 0.15 | -0.61 | 0.54 | 0.65 | 1.26 |
|  | Northern Ireland | 0.79 | 0.16 | -1.16 | 0.25 | 0.52 | 1.18 |
| Highest level of education: | |  |  |  |  |  |  |
|  | A-Level | 0.72 | 0.07 | -3.30 | 0.00 | 0.59 | 0.87 |
|  | GCSE | 0.75 | 0.08 | -2.59 | 0.01 | 0.60 | 0.93 |
|  | Other | 0.61 | 0.13 | -2.38 | 0.02 | 0.40 | 0.92 |
|  | None | 0.46 | 0.17 | -2.14 | 0.03 | 0.23 | 0.94 |
| Self-reported financial conditions: bad | | 2.57 | 0.23 | 10.35 | 0.00 | 2.15 | 3.07 |
| Health condition: yes | | 2.28 | 0.20 | 9.22 | 0.00 | 1.92 | 2.72 |
| Company size: | |  |  |  |  |  |  |
|  | 25-199 | 0.85 | 0.09 | -1.60 | 0.11 | 0.69 | 1.04 |
|  | More than 200 | 0.88 | 0.09 | -1.25 | 0.21 | 0.72 | 1.07 |
| Wave Number | |  |  |  |  |  |  |
|  | Wave 10 | 0.89 | 0.09 | -1.15 | 0.25 | 0.72 | 1.09 |
|  | Wave 11 | 1.31 | 0.15 | 2.46 | 0.01 | 1.06 | 1.63 |
|  | COVID-19 Sweeps |  |  |  |  |  |  |
|  | Apr-20 | 2.17 | 0.27 | 6.16 | 0.00 | 1.70 | 2.78 |
|  | May-20 | 1.72 | 0.22 | 4.35 | 0.00 | 1.35 | 2.20 |
|  | Jun-20 | 1.53 | 0.20 | 3.19 | 0.00 | 1.18 | 1.98 |
|  | Jul-20 | 0.97 | 0.12 | -0.27 | 0.79 | 0.75 | 1.24 |
|  | Sep-20 | 1.01 | 0.14 | 0.11 | 0.92 | 0.78 | 1.32 |
|  | Nov-20 | 1.80 | 0.26 | 4.04 | 0.00 | 1.35 | 2.38 |
|  | Jan-21 | 1.58 | 0.21 | 3.36 | 0.00 | 1.21 | 2.06 |
|  | Mar-21 | 1.32 | 0.18 | 2.08 | 0.04 | 1.02 | 1.72 |
|  | Sep-21 | 1.00 | (omitted) |  |  |  |  |
| Constant |  | 0.03 | 0.02 | -6.31 | 0.00 | 0.01 | 0.08 |

Note: Observations=29,513; Individuals=4,116 (Minimum=1; Average= 7.2; Maximum=12). **Exposure is in bold letters.** Reference categories: gender (ref.: female), age, age square, ethnicity (ref.: white), Interaction : ref (pre-pandemic-No presence of trade union), industrial group (ref.: hotel and accommodation), UK country of residence (ref.: England), education (ref.: university degree), financial situation (ref.: Good financial situation), health condition (ref.: Not having longstanding illness or impairment) and size of workplace (ref.:1 to 34 employees).

## S4.4. Union presence – adjusted model (3-way interaction), excluding public workplaces

|  |  | **Adjusted model (weighted) -- three-ways interaction** | | | | | |
| --- | --- | --- | --- | --- | --- | --- | --- |
|  |  | **OR** | **std. err.** | **z** | **P>z** | **[95% conf.** | **interval]** |
| Sex |  | 2.28 | 0.20 | 9.58 | 0.00 | 1.93 | 2.70 |
| Age | | 1.02 | 0.02 | 0.86 | 0.39 | 0.97 | 1.07 |
| Age square | | 1.00 | 0.00 | -2.05 | 0.04 | 1.00 | 1.00 |
| Ethnicity | | 0.93 | 0.16 | -0.42 | 0.68 | 0.67 | 1.29 |
| **During pandemic: 1 # Industry:** |  |  |  |  |  |  |  |
|  | **Pre-Pandemic-#Mining, Energy and Water Supply** | 3.31 | 1.95 | 2.04 | 0.04 | 1.05 | 10.50 |
|  | **Post-Pandemic-#Mining, Energy and Water Supply** | 2.51 | 0.86 | 2.70 | 0.01 | 1.29 | 4.89 |
|  | **Pre-Pandemic-#Manufacturing** | 0.86 | 0.22 | -0.60 | 0.55 | 0.52 | 1.42 |
|  | **Post-Pandemic-#Manufacturing** | 1.49 | 0.41 | 1.48 | 0.14 | 0.88 | 2.54 |
|  | **Pre-Pandemic-#Construction** | 0.70 | 0.23 | -1.08 | 0.28 | 0.36 | 1.34 |
|  | **Post-Pandemic-#Construction** | 1.27 | 0.37 | 0.81 | 0.42 | 0.72 | 2.24 |
|  | **Pre-Pandemic-#Wholesale and Retail Trade Motor Repair** | 1.15 | 0.31 | 0.52 | 0.60 | 0.68 | 1.96 |
|  | **Post-Pandemic-#Wholesale and Retail Trade Motor Repair** | 1.80 | 0.51 | 2.09 | 0.04 | 1.04 | 3.12 |
|  | **Pre-Pandemic-#Transportation and Storage** | 1.16 | 0.44 | 0.39 | 0.70 | 0.55 | 2.44 |
|  | **Post-Pandemic-#Transportation and Storage** | 1.06 | 0.37 | 0.17 | 0.87 | 0.53 | 2.10 |
|  | **Pre-Pandemic-#Accommodation and Food Services** | 1.56 | 0.53 | 1.31 | 0.19 | 0.80 | 3.03 |
|  | **Post-Pandemic-#Accommodation and Food Services** | 1.84 | 0.60 | 1.85 | 0.06 | 0.96 | 3.50 |
|  | **Pre-Pandemic-#Information and Communication** | 0.80 | 0.30 | -0.60 | 0.55 | 0.38 | 1.67 |
|  | **Post-Pandemic-#Information and Communication** | 1.81 | 0.47 | 2.26 | 0.02 | 1.08 | 3.02 |
|  | **Pre-Pandemic-#Financial and Insurance Activities** | 1.43 | 0.45 | 1.12 | 0.26 | 0.76 | 2.67 |
|  | **Post-Pandemic-#Financial and Insurance Activities** | 1.18 | 0.34 | 0.59 | 0.56 | 0.68 | 2.06 |
|  | **Pre-Pandemic-#Real Estate Activities** | 1.08 | 0.50 | 0.18 | 0.86 | 0.44 | 2.66 |
|  | **Post-Pandemic-#Real Estate Activities** | 1.81 | 0.78 | 1.39 | 0.17 | 0.78 | 4.21 |
|  | **Pre-Pandemic-#Professional Scientific and Technical** | 0.86 | 0.21 | -0.60 | 0.55 | 0.53 | 1.40 |
|  | **Post-Pandemic-#Professional Scientific and Technical** | 1.53 | 0.38 | 1.74 | 0.08 | 0.95 | 2.49 |
|  | **Pre-Pandemic-#Administrative and Support Services** | 0.67 | 0.25 | -1.10 | 0.27 | 0.32 | 1.37 |
|  | **Post-Pandemic-#Administrative and Support Services** | 1.42 | 0.43 | 1.15 | 0.25 | 0.78 | 2.58 |
|  | **Pre-Pandemic-#Public Administration and Defence** | 1.98 | 1.75 | 0.77 | 0.44 | 0.35 | 11.23 |
|  | **Post-Pandemic-#Public Administration and Defence** | 1.15 | 0.52 | 0.31 | 0.76 | 0.48 | 2.77 |
|  | **Pre-Pandemic-#Education** | 0.57 | 0.27 | -1.19 | 0.23 | 0.23 | 1.44 |
|  | **Post-Pandemic-#Education** | 1.55 | 0.48 | 1.41 | 0.16 | 0.84 | 2.85 |
|  | **Post-Pandemic-#Human Health and Social Work Activities** | 1.53 | 0.35 | 1.85 | 0.07 | 0.97 | 2.41 |
|  | **Pre-Pandemic-#Other Services** | 1.21 | 0.41 | 0.56 | 0.58 | 0.62 | 2.34 |
|  | **Post-Pandemic-#Other Services** | 1.45 | 0.36 | 1.50 | 0.13 | 0.89 | 2.34 |
| **Union presence: yes** | | 1.18 | 0.37 | 0.52 | 0.61 | 0.63 | 2.19 |
| **During pandemic: 1 # Industry # Union presence** | |  |  |  |  |  |  |
|  | **Pre-Pandemic-#Mining, Energy and Water Supply#Yes** | 0.36 | 0.29 | -1.26 | 0.21 | 0.07 | 1.77 |
|  | **Post-Pandemic-#Mining, Energy and Water Supply#Yes** | 0.57 | 0.30 | -1.07 | 0.29 | 0.21 | 1.59 |
|  | **Pre-Pandemic-#Manufacturing#Yes** | 0.62 | 0.29 | -1.01 | 0.31 | 0.25 | 1.56 |
|  | **Post-Pandemic-#Manufacturing#Yes** | 0.65 | 0.29 | -0.98 | 0.33 | 0.27 | 1.55 |
|  | **Pre-Pandemic-#Construction#Yes** | 2.13 | 1.45 | 1.11 | 0.27 | 0.56 | 8.13 |
|  | **Post-Pandemic-#Construction#Yes** | 1.11 | 0.58 | 0.20 | 0.85 | 0.40 | 3.09 |
|  | **Pre-Pandemic-#Wholesale and Retail Trade Motor Repair#Yes** | 0.92 | 0.40 | -0.20 | 0.84 | 0.39 | 2.14 |
|  | **Post-Pandemic-#Wholesale and Retail Trade Motor Repair#Yes** | 0.70 | 0.29 | -0.87 | 0.39 | 0.31 | 1.57 |
|  | **Pre-Pandemic-#Transportation and Storage#Yes** | 0.68 | 0.36 | -0.73 | 0.46 | 0.24 | 1.92 |
|  | **Post-Pandemic-#Transportation and Storage#Yes** | 1.30 | 0.64 | 0.54 | 0.59 | 0.50 | 3.41 |
|  | **Pre-Pandemic-#Accommodation and Food Services#Yes** | 0.24 | 0.19 | -1.77 | 0.08 | 0.05 | 1.16 |
|  | **Post-Pandemic-#Accommodation and Food Services#Yes** | 0.42 | 0.26 | -1.43 | 0.15 | 0.13 | 1.38 |
|  | **Pre-Pandemic-#Information and Communication#Yes** | 1.98 | 1.19 | 1.13 | 0.26 | 0.60 | 6.46 |
|  | **Post-Pandemic-#Information and Communication#Yes** | 0.45 | 0.24 | -1.47 | 0.14 | 0.15 | 1.30 |
|  | **Pre-Pandemic-#Financial and Insurance Activities#Yes** | 0.54 | 0.29 | -1.17 | 0.24 | 0.19 | 1.53 |
|  | **Post-Pandemic-#Financial and Insurance Activities#Yes** | 0.67 | 0.31 | -0.86 | 0.39 | 0.27 | 1.66 |
|  | **Pre-Pandemic-#Real Estate Activities#Yes** | 4.08 | 3.05 | 1.88 | 0.06 | 0.94 | 17.65 |
|  | **Post-Pandemic-#Real Estate Activities#Yes** | 0.78 | 0.59 | -0.33 | 0.74 | 0.17 | 3.45 |
|  | **Pre-Pandemic-#Professional Scientific and Technical#Yes** | 1.00 | 0.50 | 0.00 | 1.00 | 0.37 | 2.68 |
|  | **Post-Pandemic-#Professional Scientific and Technical#Yes** | 1.25 | 0.54 | 0.52 | 0.60 | 0.54 | 2.92 |
|  | **Pre-Pandemic-#Administrative and Support Services#Yes** | 1.00 | 0.76 | 0.00 | 1.00 | 0.23 | 4.40 |
|  | **Post-Pandemic-#Administrative and Support Services#Yes** | 0.71 | 0.43 | -0.56 | 0.57 | 0.22 | 2.30 |
|  | **Pre-Pandemic-#Public Administration and Defence#Yes** | 0.93 | 0.94 | -0.07 | 0.94 | 0.13 | 6.73 |
|  | **Post-Pandemic-#Public Administration and Defence#Yes** | 1.91 | 1.14 | 1.08 | 0.28 | 0.59 | 6.17 |
|  | **Pre-Pandemic-#Education#Yes** | 1.53 | 0.88 | 0.75 | 0.45 | 0.50 | 4.69 |
|  | **Post-Pandemic-#Education#Yes** | 1.20 | 0.53 | 0.41 | 0.68 | 0.50 | 2.85 |
|  | **Post-Pandemic-#Human Health and Social Work Activities#Yes** | 0.85 | 0.36 | -0.38 | 0.71 | 0.38 | 1.93 |
|  | **Pre-Pandemic-#Other Services #Yes** | 0.56 | 0.37 | -0.88 | 0.38 | 0.15 | 2.04 |
|  | **Post-Pandemic-#Other Services #Yes** | 0.52 | 0.23 | -1.48 | 0.14 | 0.22 | 1.24 |
| Country: | |  |  |  |  |  |  |
|  | Wales | 1.31 | 0.23 | 1.54 | 0.12 | 0.93 | 1.86 |
|  | Scotland | 0.90 | 0.15 | -0.63 | 0.53 | 0.64 | 1.26 |
|  | Northern Ireland | 0.79 | 0.17 | -1.14 | 0.26 | 0.52 | 1.19 |
| Highest level of education: | |  |  |  |  |  |  |
|  | A-Level | 0.72 | 0.07 | -3.16 | 0.00 | 0.59 | 0.88 |
|  | GCSE | 0.76 | 0.09 | -2.43 | 0.02 | 0.61 | 0.95 |
|  | Other | 0.62 | 0.13 | -2.26 | 0.02 | 0.41 | 0.94 |
|  | None | 0.49 | 0.18 | -1.97 | 0.05 | 0.24 | 1.00 |
| Self-reported financial conditions: bad | | 2.55 | 0.23 | 10.20 | 0.00 | 2.13 | 3.05 |
| Health condition: yes | | 2.29 | 0.21 | 9.22 | 0.00 | 1.92 | 2.73 |
| Company size: | |  |  |  |  |  |  |
|  | 25-199 | 0.85 | 0.09 | -1.62 | 0.11 | 0.69 | 1.04 |
|  | More than 200 | 0.88 | 0.09 | -1.23 | 0.22 | 0.72 | 1.08 |
| Wave Number | |  |  |  |  |  |  |
|  | Wave 10 | 0.88 | 0.09 | -1.19 | 0.23 | 0.72 | 1.08 |
|  | Wave 11 | 1.32 | 0.15 | 2.55 | 0.01 | 1.07 | 1.64 |
|  | **COVID-19 Sweeps** |  |  |  |  |  |  |
|  | Apr-20 | 2.17 | 0.27 | 6.12 | 0.00 | 1.69 | 2.77 |
|  | May-20 | 1.71 | 0.21 | 4.30 | 0.00 | 1.34 | 2.19 |
|  | Jun-20 | 1.53 | 0.20 | 3.20 | 0.00 | 1.18 | 1.99 |
|  | Jul-20 | 0.96 | 0.12 | -0.31 | 0.76 | 0.75 | 1.23 |
|  | Sep-20 | 1.01 | 0.14 | 0.06 | 0.95 | 0.77 | 1.32 |
|  | Nov-20 | 1.79 | 0.26 | 4.01 | 0.00 | 1.35 | 2.38 |
|  | Jan-21 | 1.57 | 0.21 | 3.32 | 0.00 | 1.20 | 2.04 |
|  | Mar-21 | 1.32 | 0.18 | 2.08 | 0.04 | 1.02 | 1.72 |
|  | Sep-21 | 1.00 | (omitted) |  |  |  |  |
| Constant | | 0.02 | 0.01 | -6.42 | 0.00 | 0.01 | 0.07 |

Note: Observations=29,513; Individuals=4,116 (Minimum=1; Average= 7.2; Maximum=12). **Exposure is in bold letters.** Reference categories: gender (ref.: female), age, age square, ethnicity (ref.: white), Interaction : ref (pre-pandemic-No presence of trade union-Hotel and Accommodation Industry ), UK country of residence (ref.: England), education (ref.: university degree), financial situation (ref.: Good financial situation), health condition (ref.: Not having longstanding illness or impairment) and size of workplace (ref.:1 to 34 employees),‘#’ indicates interaction effects.

## S4.5. Union presence – adjusted model (2-way interaction), GHQ-case – adjusting for trade union membership

|  |  | **Adjusted model (weighted)** | | | | | |
| --- | --- | --- | --- | --- | --- | --- | --- |
|  |  | **OR.** | **Robust std. err.** | **z** | **P>z** | **[95% conf.** | **interval]** |
| sex |  | 2.16 | 0.15 | 11.44 | 0.00 | 1.90 | 2.47 |
| age |  | 1.00 | 0.02 | 0.11 | 0.91 | 0.97 | 1.04 |
| age_square | | 1.00 | 0.00 | -1.39 | 0.16 | 1.00 | 1.00 |
| Ethnicity | | 0.90 | 0.11 | -0.91 | 0.36 | 0.71 | 1.13 |
| **During pandemic: 1** | | **1.46** | **0.16** | **3.40** | **0.00** | **1.17** | **1.81** |
| **Union presence: yes** | | **1.04** | **0.10** | **0.39** | **0.70** | **0.85** | **1.27** |
| **During pandemic: 1 # Union presence: yes** | | **0.89** | **0.09** | **-1.17** | **0.24** | **0.74** | **1.08** |
| Industry: | |  |  |  |  |  |  |
|  | Mining, Energy and Water Supply | 1.13 | 0.29 | 0.48 | 0.63 | 0.68 | 1.87 |
|  | Manufacturing | 0.66 | 0.13 | -2.12 | 0.03 | 0.45 | 0.97 |
|  | Construction | 0.68 | 0.15 | -1.73 | 0.08 | 0.44 | 1.05 |
|  | Wholesale and Retail Trade Motor Repair | 0.85 | 0.16 | -0.86 | 0.39 | 0.59 | 1.23 |
|  | Transportation and Storage | 0.68 | 0.15 | -1.81 | 0.07 | 0.44 | 1.03 |
|  | Information and Communication | 0.86 | 0.18 | -0.76 | 0.45 | 0.57 | 1.28 |
|  | Financial and Insurance Activities | 0.65 | 0.14 | -1.96 | 0.05 | 0.42 | 1.00 |
|  | Real Estate Activities | 0.96 | 0.27 | -0.13 | 0.90 | 0.56 | 1.66 |
|  | Professional Scientific and Technical | 0.80 | 0.15 | -1.21 | 0.23 | 0.55 | 1.15 |
|  | Administrative and Support Services | 0.62 | 0.13 | -2.26 | 0.02 | 0.41 | 0.94 |
|  | Public Administration and Defence | 0.75 | 0.14 | -1.50 | 0.13 | 0.52 | 1.09 |
|  | Education | 0.78 | 0.14 | -1.41 | 0.16 | 0.55 | 1.10 |
|  | Human Health and Social Work Activities | 0.80 | 0.14 | -1.24 | 0.21 | 0.57 | 1.14 |
|  | Other Services | 0.67 | 0.13 | -2.11 | 0.04 | 0.47 | 0.97 |
| Trade union membership | Yes: | 1.15 | 0.10 | 1.64 | 0.10 | 0.97 | 1.35 |
| Country: | |  |  |  |  |  |  |
|  | Wales | 1.23 | 0.16 | 1.65 | 0.10 | 0.96 | 1.59 |
|  | Scotland | 0.99 | 0.11 | -0.10 | 0.92 | 0.80 | 1.23 |
|  | Northern Ireland | 0.86 | 0.13 | -1.02 | 0.31 | 0.64 | 1.15 |
| Highest level of education: | |  |  |  |  |  |  |
|  | A-Level | 0.73 | 0.06 | -3.90 | 0.00 | 0.63 | 0.86 |
|  | GCSE | 0.76 | 0.07 | -3.13 | 0.00 | 0.64 | 0.90 |
|  | Other | 0.55 | 0.09 | -3.46 | 0.00 | 0.39 | 0.77 |
|  | None | 0.37 | 0.12 | -3.14 | 0.00 | 0.20 | 0.69 |
| Self-reported financial conditions: bad | | 2.44 | 0.16 | 13.45 | 0.00 | 2.14 | 2.78 |
| Health condition: yes | | 2.09 | 0.14 | 11.20 | 0.00 | 1.83 | 2.37 |
| Company size: | |  |  |  |  |  |  |
|  | 25-199 | 0.88 | 0.07 | -1.55 | 0.12 | 0.76 | 1.03 |
|  | More than 200 | 0.94 | 0.07 | -0.73 | 0.47 | 0.81 | 1.10 |
| Wave Number | |  |  |  |  |  |  |
|  | Wave 10 | 0.92 | 0.07 | -1.13 | 0.26 | 0.78 | 1.07 |
|  | Wave 11 | 1.23 | 0.11 | 2.36 | 0.02 | 1.04 | 1.45 |
|  | COVID-19 Sweeps |  |  |  |  |  |  |
|  | Apr-20 | 0.92 | 0.07 | -1.13 | 0.26 | 0.78 | 1.07 |
|  | May-20 | 1.23 | 0.11 | 2.36 | 0.02 | 1.04 | 1.45 |
|  | Jun-20 | 2.28 | 0.20 | 9.18 | 0.00 | 1.91 | 2.72 |
|  | Jul-20 | 1.81 | 0.17 | 6.46 | 0.00 | 1.51 | 2.16 |
|  | Sep-20 | 1.62 | 0.16 | 4.99 | 0.00 | 1.34 | 1.96 |
|  | Nov-20 | 0.96 | 0.09 | -0.45 | 0.65 | 0.80 | 1.15 |
|  | Jan-21 | 1.04 | 0.10 | 0.41 | 0.68 | 0.86 | 1.26 |
|  | Mar-21 | 1.81 | 0.18 | 5.80 | 0.00 | 1.48 | 2.21 |
|  | Sep-21 | 1.78 | 0.17 | 6.00 | 0.00 | 1.48 | 2.15 |
| Constant |  | 0.05 | 0.02 | -6.86 | 0.00 | 0.02 | 0.11 |

Note: Observations=49,768; Individuals=5,984 (Minimum=1; Average= 8.3; Maximum=12). **Exposure is in bold letters.** Reference categories: gender (ref.: female), age, age square, ethnicity (ref.: white), Interaction : ref (pre-pandemic-No presence of trade union), industrial group (ref.: hotel and accommodation), UK country of residence (ref.: England), education (ref.: university degree), financial situation (ref.: Good financial situation), health condition (ref.: Not having longstanding illness or impairment) and size of workplace (ref.:1 to 34 employees)

## S4.6. Union presence – adjusted model (2-way interaction), GHQ-36

|  |  | **Adjusted model (weighted)** | | | | | |
| --- | --- | --- | --- | --- | --- | --- | --- |
|  |  | **Coef.** | **std. err.** | **z** | **P>z** | **[95% conf.** | **interval]** |
| sex |  | 1.07 | 0.11 | 9.45 | 0.00 | 0.85 | 1.29 |
| age |  | 0.04 | 0.03 | 1.35 | 0.18 | -0.02 | 0.10 |
| age_square | | 0.00 | 0.00 | -2.95 | 0.00 | 0.00 | 0.00 |
| Ethnicity | | -0.22 | 0.21 | -1.08 | 0.28 | -0.63 | 0.18 |
| **During pandemic: 1** | | 1.21 | 0.14 | 8.37 | 0.00 | 0.93 | 1.49 |
| **Union presence: yes** | | 0.08 | 0.12 | 0.63 | 0.53 | -0.17 | 0.32 |
| **During pandemic: 1 # Union presence: yes** | | -0.08 | 0.13 | -0.60 | 0.55 | -0.33 | 0.18 |
| Industry: | |  |  |  |  |  |  |
|  | Mining, Energy and Water Supply | 0.69 | 0.39 | 1.78 | 0.08 | -0.07 | 1.46 |
|  | Manufacturing | -0.03 | 0.32 | -0.11 | 0.92 | -0.67 | 0.60 |
|  | Construction | -0.19 | 0.36 | -0.53 | 0.60 | -0.89 | 0.51 |
|  | Wholesale and Retail Trade Motor Repair | 0.17 | 0.32 | 0.54 | 0.59 | -0.45 | 0.80 |
|  | Transportation and Storage | -0.13 | 0.34 | -0.38 | 0.70 | -0.79 | 0.53 |
|  | Information and Communication | 0.04 | 0.34 | 0.12 | 0.90 | -0.62 | 0.71 |
|  | Financial and Insurance Activities | -0.18 | 0.38 | -0.47 | 0.64 | -0.93 | 0.57 |
|  | Real Estate Activities | 0.57 | 0.48 | 1.20 | 0.23 | -0.36 | 1.50 |
|  | Professional Scientific and Technical | 0.03 | 0.31 | 0.10 | 0.92 | -0.58 | 0.64 |
|  | Administrative and Support Services | 0.11 | 0.36 | 0.30 | 0.76 | -0.60 | 0.82 |
|  | Public Administration and Defence | 0.11 | 0.31 | 0.35 | 0.72 | -0.50 | 0.73 |
|  | Education | 0.06 | 0.33 | 0.18 | 0.86 | -0.58 | 0.70 |
|  | Human Health and Social Work Activities | 0.17 | 0.30 | 0.57 | 0.57 | -0.42 | 0.77 |
|  | Other Services | -0.10 | 0.31 | -0.32 | 0.75 | -0.70 | 0.51 |
| Country: | |  |  |  |  |  |  |
|  | Wales | 0.40 | 0.23 | 1.73 | 0.08 | -0.05 | 0.86 |
|  | Scotland | -0.24 | 0.20 | -1.16 | 0.24 | -0.64 | 0.16 |
|  | Northern Ireland | -0.50 | 0.24 | -2.11 | 0.04 | -0.96 | -0.04 |
| Highest level of education: | |  |  |  |  |  |  |
|  | A-Level | -0.16 | 0.14 | -1.15 | 0.25 | -0.43 | 0.11 |
|  | GCSE | 0.04 | 0.16 | 0.26 | 0.80 | -0.27 | 0.35 |
|  | Other | -0.38 | 0.26 | -1.46 | 0.14 | -0.88 | 0.13 |
|  | None | -0.87 | 0.38 | -2.27 | 0.02 | -1.62 | -0.12 |
| Self-reported financial conditions: bad | | 1.70 | 0.12 | 14.61 | 0.00 | 1.47 | 1.93 |
| Health condition: yes | | 1.13 | 0.12 | 9.79 | 0.00 | 0.90 | 1.36 |
| Company size: | |  |  |  |  |  |  |
|  | 25-199 | 0.03 | 0.13 | 0.26 | 0.80 | -0.22 | 0.28 |
|  | More than 200 | -0.08 | 0.14 | -0.60 | 0.55 | -0.35 | 0.18 |
| Wave Number | |  |  |  |  |  |  |
|  | Wave 10 | -0.04 | 0.09 | -0.50 | 0.62 | -0.22 | 0.13 |
|  | Wave 11 | 0.35 | 0.10 | 3.40 | 0.00 | 0.15 | 0.55 |
|  | COVID-19 Sweeps |  |  |  |  |  |  |
|  | Apr-20 | 0.22 | 0.13 | 1.62 | 0.11 | -0.05 | 0.48 |
|  | May-20 | 0.22 | 0.14 | 1.55 | 0.12 | -0.06 | 0.49 |
|  | Jun-20 | 0.36 | 0.14 | 2.50 | 0.01 | 0.08 | 0.64 |
|  | Jul-20 | -0.39 | 0.14 | -2.85 | 0.00 | -0.66 | -0.12 |
|  | Sep-20 | -0.20 | 0.14 | -1.50 | 0.13 | -0.47 | 0.06 |
|  | Nov-20 | 0.81 | 0.14 | 5.76 | 0.00 | 0.53 | 1.08 |
|  | Jan-21 | 0.72 | 0.14 | 5.18 | 0.00 | 0.45 | 0.99 |
|  | Mar-21 | 0.22 | 0.13 | 1.64 | 0.10 | -0.04 | 0.48 |
|  | Sep-21 | 0.00 | (omitted) |  |  |  |  |
| Constant |  | 9.22 | 0.75 | 12.23 | 0.00 | 7.74 | 10.70 |

Note: Observations=49,915; Individuals=5,988 (Minimum=1; Average= 8.3; Maximum=12). **Exposure is in bold letters.** Reference categories: gender (ref.: female), age, age square, ethnicity (ref.: white), Interaction : ref (pre-pandemic-No presence of trade union), industrial group (ref.: hotel and accommodation), UK country of residence (ref.: England), education (ref.: university degree), financial situation (ref.: Good financial situation), health condition (ref.: Not having longstanding illness or impairment) and size of workplace (ref.:1 to 34 employees)

## S4.7. Union membership – adjusted model (2-way interaction), GHQ-36

|  |  | **Adjusted model (weighted)** | | | | | |
| --- | --- | --- | --- | --- | --- | --- | --- |
|  |  | **Coef.** | **std. err.** | **z** | **P>z** | **[95% conf.** | **interval]** |
| sex |  | 1.03 | 0.15 | 6.64 | 0.00 | 0.72 | 1.33 |
| age |  | 0.05 | 0.05 | 1.11 | 0.27 | -0.04 | 0.14 |
| age_square | | 0.00 | 0.00 | -1.87 | 0.06 | 0.00 | 0.00 |
| Ethnicity | | -0.28 | 0.26 | -1.09 | 0.28 | -0.78 | 0.22 |
| **During pandemic: 1** | | 1.14 | 0.20 | 5.82 | 0.00 | 0.76 | 1.52 |
| **Union presence: yes** | | 0.26 | 0.17 | 1.52 | 0.13 | -0.07 | 0.59 |
| **During pandemic: 1 # Union presence: yes** | | -0.30 | 0.18 | -1.68 | 0.09 | -0.66 | 0.05 |
| Industry: | |  |  |  |  |  |  |
|  | Mining, Energy and Water Supply | 0.29 | 0.55 | 0.52 | 0.60 | -0.80 | 1.37 |
|  | Manufacturing | -0.11 | 0.48 | -0.23 | 0.82 | -1.05 | 0.82 |
|  | Construction | 0.23 | 0.56 | 0.41 | 0.68 | -0.86 | 1.32 |
|  | Wholesale and Retail Trade Motor Repair | 0.46 | 0.47 | 0.98 | 0.33 | -0.46 | 1.38 |
|  | Transportation and Storage | 0.19 | 0.49 | 0.38 | 0.70 | -0.77 | 1.14 |
|  | Information and Communication | 0.12 | 0.53 | 0.22 | 0.83 | -0.92 | 1.15 |
|  | Financial and Insurance Activities | 0.04 | 0.52 | 0.07 | 0.95 | -0.99 | 1.06 |
|  | Real Estate Activities | 0.87 | 0.58 | 1.52 | 0.13 | -0.25 | 2.00 |
|  | Professional Scientific and Technical | 0.27 | 0.48 | 0.56 | 0.57 | -0.67 | 1.21 |
|  | Administrative and Support Services | 0.27 | 0.52 | 0.52 | 0.60 | -0.75 | 1.30 |
|  | Public Administration and Defence | 0.36 | 0.45 | 0.81 | 0.42 | -0.52 | 1.25 |
|  | Education | 0.31 | 0.45 | 0.69 | 0.49 | -0.57 | 1.20 |
|  | Human Health and Social Work Activities | 0.39 | 0.45 | 0.86 | 0.39 | -0.49 | 1.27 |
|  | Other Services | 0.15 | 0.47 | 0.32 | 0.75 | -0.77 | 1.06 |
| Country: | |  |  |  |  |  |  |
|  | Wales | 0.22 | 0.29 | 0.74 | 0.46 | -0.36 | 0.79 |
|  | Scotland | -0.31 | 0.22 | -1.42 | 0.16 | -0.75 | 0.12 |
|  | Northern Ireland | -0.48 | 0.30 | -1.61 | 0.11 | -1.08 | 0.11 |
| Highest level of education: | |  |  |  |  |  |  |
|  | A-Level | -0.27 | 0.19 | -1.40 | 0.16 | -0.64 | 0.11 |
|  | GCSE | -0.22 | 0.22 | -1.03 | 0.31 | -0.64 | 0.20 |
|  | Other | -0.92 | 0.33 | -2.75 | 0.01 | -1.57 | -0.26 |
|  | None | -0.91 | 0.55 | -1.66 | 0.10 | -1.98 | 0.17 |
| Self-reported financial conditions: bad | | 1.83 | 0.15 | 12.06 | 0.00 | 1.53 | 2.13 |
| Health condition: yes | | 0.97 | 0.16 | 6.14 | 0.00 | 0.66 | 1.28 |
| Company size: | |  |  |  |  |  |  |
|  | 25-199 | 0.05 | 0.21 | 0.23 | 0.82 | -0.37 | 0.47 |
|  | More than 200 | 0.14 | 0.21 | 0.65 | 0.51 | -0.28 | 0.56 |
| Wave Number | |  |  |  |  |  |  |
|  | Wave 10 | 0.89 | 0.09 | -1.12 | 0.26 | 0.72 | 1.09 |
|  | Wave 11 | 1.35 | 0.16 | 2.51 | 0.01 | 1.07 | 1.70 |
|  | COVID-19 Sweeps |  |  |  |  |  |  |
|  | Apr-20 | -0.15 | 0.13 | -1.20 | 0.23 | -0.40 | 0.10 |
|  | May-20 | 0.32 | 0.15 | 2.11 | 0.04 | 0.02 | 0.62 |
|  | Jun-20 | 0.38 | 0.17 | 2.22 | 0.03 | 0.04 | 0.71 |
|  | Jul-20 | 0.37 | 0.17 | 2.18 | 0.03 | 0.04 | 0.70 |
|  | Sep-20 | 0.44 | 0.18 | 2.42 | 0.02 | 0.08 | 0.79 |
|  | Nov-20 | -0.40 | 0.17 | -2.34 | 0.02 | -0.74 | -0.06 |
|  | Jan-21 | -0.16 | 0.18 | -0.89 | 0.38 | -0.50 | 0.19 |
|  | Mar-21 | 0.93 | 0.18 | 5.07 | 0.00 | 0.57 | 1.29 |
|  | Sep-21 | 0.00 | (omitted) |  |  |  |  |
| Constant |  | 8.58 | 1.14 | 7.49 | 0.00 | 6.33 | 10.82 |

Note: Observations=27,971; Individuals=3,341 (Minimum=1; Average= 8.4; Maximum=12). **Exposure is in bold letters.** Reference categories: gender (ref.: female), age, age square, ethnicity (ref.: white), Interaction : ref (pre-pandemic-No presence of trade union), industrial group (ref.: hotel and accommodation), UK country of residence (ref.: England), education (ref.: university degree), financial situation (ref.: Good financial situation), health condition (ref.: Not having longstanding illness or impairment) and size of workplace (ref.:1 to 34 employees)

## S4.8. Union presence – adjusted model (2-way interaction)- Removing Financial situation from confounders’ list

|  |  | **Adjusted model (Unweighted)** | | | | | |
| --- | --- | --- | --- | --- | --- | --- | --- |
|  |  | **OR** | **Robust std. err.** | **z** | **P>z** | **[95% conf.** | **interval]** |
| Sex |  | 2.19 | 0.15 | 11.38 | 0.00 | 1.91 | 2.51 |
| Age |  | 1.02 | 0.02 | 0.98 | 0.33 | 0.98 | 1.06 |
| Age_square | | 1.00 | 0.00 | -2.28 | 0.02 | 1.00 | 1.00 |
| Ethnicity | | 1.02 | 0.13 | 0.13 | 0.89 | 0.80 | 1.30 |
| **During pandemic: 1** | | **1.42** | **0.16** | **3.19** | **0.00** | **1.15** | **1.76** |
| **Union presence: yes** | | **1.13** | **0.10** | **1.35** | **0.18** | **0.95** | **1.35** |
| **During pandemic: 1 # Union presence: yes** | | **0.86** | **0.08** | **-1.59** | **0.11** | **0.71** | **1.04** |
| Industry: | |  |  |  |  |  |  |
|  | Mining, Energy and Water Supply | 1.00 | 0.25 | 0.00 | 1.00 | 0.61 | 1.65 |
|  | Manufacturing | 0.60 | 0.12 | -2.65 | 0.01 | 0.41 | 0.87 |
|  | Construction | 0.59 | 0.13 | -2.31 | 0.02 | 0.38 | 0.92 |
|  | Wholesale and Retail Trade Motor Repair | 0.80 | 0.15 | -1.18 | 0.24 | 0.55 | 1.16 |
|  | Transportation and Storage | 0.60 | 0.13 | -2.31 | 0.02 | 0.39 | 0.93 |
|  | Information and Communication | 0.78 | 0.16 | -1.22 | 0.22 | 0.52 | 1.16 |
|  | Financial and Insurance Activities | 0.56 | 0.12 | -2.60 | 0.01 | 0.36 | 0.87 |
|  | Real Estate Activities | 0.85 | 0.24 | -0.58 | 0.56 | 0.49 | 1.46 |
|  | Professional Scientific and Technical | 0.70 | 0.13 | -1.94 | 0.05 | 0.49 | 1.00 |
|  | Administrative and Support Services | 0.58 | 0.12 | -2.68 | 0.01 | 0.39 | 0.86 |
|  | Public Administration and Defence | 0.68 | 0.13 | -2.04 | 0.04 | 0.47 | 0.99 |
|  | Education | 0.72 | 0.13 | -1.84 | 0.07 | 0.51 | 1.02 |
|  | Human Health and Social Work Activities | 0.75 | 0.13 | -1.60 | 0.11 | 0.53 | 1.07 |
|  | Other Services | 0.62 | 0.12 | -2.59 | 0.01 | 0.43 | 0.89 |
| Country: | |  |  |  |  |  |  |
|  | Wales | 1.27 | 0.17 | 1.81 | 0.07 | 0.98 | 1.64 |
|  | Scotland | 1.00 | 0.11 | 0.02 | 0.99 | 0.80 | 1.25 |
|  | Northern Ireland | 0.88 | 0.13 | -0.86 | 0.39 | 0.65 | 1.18 |
| Highest level of education: | |  |  |  |  |  |  |
|  | A-Level | 0.78 | 0.06 | -3.10 | 0.00 | 0.66 | 0.91 |
|  | GCSE | 0.85 | 0.08 | -1.82 | 0.07 | 0.71 | 1.01 |
|  | Other | 0.59 | 0.10 | -3.03 | 0.00 | 0.42 | 0.83 |
|  | None | 0.44 | 0.14 | -2.55 | 0.01 | 0.24 | 0.83 |
| Health condition: yes (having longstanding illness or impairment) | | 2.16 | 0.14 | 11.63 | 0.00 | 1.90 | 2.47 |
| Company size: | |  |  |  |  |  |  |
|  | 25-199 | 0.88 | 0.07 | -1.60 | 0.11 | 0.75 | 1.03 |
|  | More than 200 | 0.91 | 0.07 | -1.14 | 0.26 | 0.78 | 1.07 |
| Wave Number | |  |  |  |  |  |  |
|  | Wave 10 | 0.91 | 0.07 | -1.14 | 0.25 | 0.78 | 1.07 |
|  | Wave 11 | 1.23 | 0.11 | 2.43 | 0.02 | 1.04 | 1.46 |
|  | **COVID-19 sweeps** |  |  |  |  |  |  |
|  | Apr-20 | 2.26 | 0.20 | 9.02 | 0.00 | 1.89 | 2.69 |
|  | May-20 | 1.78 | 0.16 | 6.30 | 0.00 | 1.49 | 2.13 |
|  | Jun-20 | 1.60 | 0.16 | 4.82 | 0.00 | 1.32 | 1.93 |
|  | Jul-20 | 0.97 | 0.09 | -0.37 | 0.71 | 0.81 | 1.16 |
|  | Sep-20 | 1.04 | 0.10 | 0.43 | 0.67 | 0.86 | 1.26 |
|  | Nov-20 | 1.82 | 0.18 | 5.96 | 0.00 | 1.50 | 2.22 |
|  | Jan-21 | 1.79 | 0.17 | 6.14 | 0.00 | 1.49 | 2.16 |
|  | Mar-21 | 1.23 | 0.12 | 2.20 | 0.03 | 1.02 | 1.49 |
|  | Sep-21 | 1.00 | (omitted) |  |  |  |  |
| Constant | | 0.04 | 0.02 | -7.19 | 0.00 | 0.02 | 0.09 |

Note: Observations=49,915; Individuals=5,988 (Minimum=1; Average= 8.3; Maximum=12). **Exposure is in bold letters.** Reference categories: gender (ref.: female), age, age square, ethnicity (ref.: white), Interaction : ref (pre-pandemic-No presence of trade union), industrial group (ref.: hotel and accommodation), UK country of residence (ref.: England), education (ref.: university degree), financial situation (ref.: Good financial situation), health condition (ref.: Not having longstanding illness or impairment) and size of workplace (ref.:1 to 34 employees)

## S4.9. Union presence – adjusted model (2-way interaction)- Unweighted estimates

|  |  | **Adjusted model (Unweighted)** | | | | | |
| --- | --- | --- | --- | --- | --- | --- | --- |
|  |  | **OR** | **Robust std. err.** | **z** | **P>z** | **[95% conf.** | **interval]** |
| Sex |  | 2.01 | 0.13 | 11.11 | 0.00 | 1.78 | 2.28 |
| Age |  | 1.00 | 0.02 | -0.09 | 0.93 | 0.97 | 1.03 |
| Age_square | | 1.00 | 0.00 | -1.49 | 0.14 | 1.00 | 1.00 |
| Ethnicity | | 0.84 | 0.09 | -1.63 | 0.10 | 0.68 | 1.04 |
| **During pandemic: 1** | | **1.46** | **0.13** | **4.35** | **0.00** | **1.23** | **1.74** |
| **Union presence: yes** | | **1.08** | **0.09** | **1.02** | **0.31** | **0.93** | **1.26** |
| **During pandemic: 1 # Union presence: yes** | | **0.93** | **0.08** | **-0.91** | **0.37** | **0.79** | **1.09** |
| Industry: | |  |  |  |  |  |  |
|  | Mining, Energy and Water Supply | 1.11 | 0.23 | 0.47 | 0.64 | 0.73 | 1.67 |
|  | Manufacturing | 0.75 | 0.12 | -1.75 | 0.08 | 0.54 | 1.03 |
|  | Construction | 0.73 | 0.14 | -1.70 | 0.09 | 0.51 | 1.05 |
|  | Wholesale and Retail Trade Motor Repair | 0.85 | 0.12 | -1.14 | 0.26 | 0.63 | 1.13 |
|  | Transportation and Storage | 0.84 | 0.15 | -1.01 | 0.31 | 0.59 | 1.18 |
|  | Information and Communication | 1.01 | 0.17 | 0.06 | 0.95 | 0.73 | 1.40 |
|  | Financial and Insurance Activities | 0.72 | 0.13 | -1.79 | 0.07 | 0.50 | 1.03 |
|  | Real Estate Activities | 1.06 | 0.25 | 0.24 | 0.81 | 0.66 | 1.70 |
|  | Professional Scientific and Technical | 0.93 | 0.14 | -0.47 | 0.64 | 0.69 | 1.26 |
|  | Administrative and Support Services | 0.74 | 0.12 | -1.92 | 0.06 | 0.54 | 1.01 |
|  | Public Administration and Defence | 0.86 | 0.13 | -1.01 | 0.31 | 0.63 | 1.16 |
|  | Education | 0.87 | 0.13 | -0.99 | 0.32 | 0.65 | 1.15 |
|  | Human Health and Social Work Activities | 0.92 | 0.13 | -0.56 | 0.58 | 0.70 | 1.22 |
|  | Other Services | 0.86 | 0.13 | -1.01 | 0.32 | 0.65 | 1.15 |
| Country: | |  |  |  |  |  |  |
|  | Wales | 1.11 | 0.14 | 0.86 | 0.39 | 0.87 | 1.41 |
|  | Scotland | 0.95 | 0.09 | -0.56 | 0.57 | 0.78 | 1.15 |
|  | Northern Ireland | 0.84 | 0.11 | -1.28 | 0.20 | 0.64 | 1.10 |
| Highest level of education: | |  |  |  |  |  |  |
|  | A-Level | 0.75 | 0.06 | -3.83 | 0.00 | 0.65 | 0.87 |
|  | GCSE | 0.78 | 0.07 | -2.99 | 0.00 | 0.66 | 0.92 |
|  | Other | 0.61 | 0.10 | -3.00 | 0.00 | 0.45 | 0.84 |
|  | None | 0.43 | 0.13 | -2.85 | 0.00 | 0.24 | 0.77 |
| Self-reported financial conditions: bad | | 2.54 | 0.12 | 19.46 | 0.00 | 2.31 | 2.79 |
| Health condition: yes (having longstanding illness or impairment) | | 2.05 | 0.12 | 12.56 | 0.00 | 1.83 | 2.29 |
| Company size: | |  |  |  |  |  |  |
|  | 25-199 | 0.91 | 0.06 | -1.40 | 0.16 | 0.79 | 1.04 |
|  | More than 200 | 0.91 | 0.06 | -1.41 | 0.16 | 0.79 | 1.04 |
| Wave Number | |  |  |  |  |  |  |
|  | Wave 10 | 0.98 | 0.06 | -0.31 | 0.76 | 0.86 | 1.11 |
|  | Wave 11 | 1.22 | 0.09 | 2.76 | 0.01 | 1.06 | 1.41 |
|  | **COVID-19 sweeps** |  |  |  |  |  |  |
|  | Apr-20 | 2.23 | 0.15 | 11.74 | 0.00 | 1.95 | 2.55 |
|  | May-20 | 1.76 | 0.12 | 8.17 | 0.00 | 1.54 | 2.01 |
|  | Jun-20 | 1.50 | 0.10 | 5.80 | 0.00 | 1.31 | 1.72 |
|  | Jul-20 | 0.92 | 0.07 | -1.10 | 0.27 | 0.80 | 1.06 |
|  | Sep-20 | 1.03 | 0.08 | 0.37 | 0.71 | 0.89 | 1.19 |
|  | Nov-20 | 1.70 | 0.12 | 7.57 | 0.00 | 1.48 | 1.95 |
|  | Jan-21 | 1.83 | 0.13 | 8.44 | 0.00 | 1.59 | 2.10 |
|  | Mar-21 | 1.28 | 0.09 | 3.65 | 0.00 | 1.12 | 1.47 |
|  | Sep-21 | 1.00 | (omitted) |  |  |  |  |
| Constant | | 0.06 | 0.02 | -7.06 | 0.00 | 0.03 | 0.13 |

## S4.10. Marginal means for GHQ-caseness by union presence controlling for union membership and marginal means of GHQ-caseness by union membership controlling for union presence

**B**

**A**


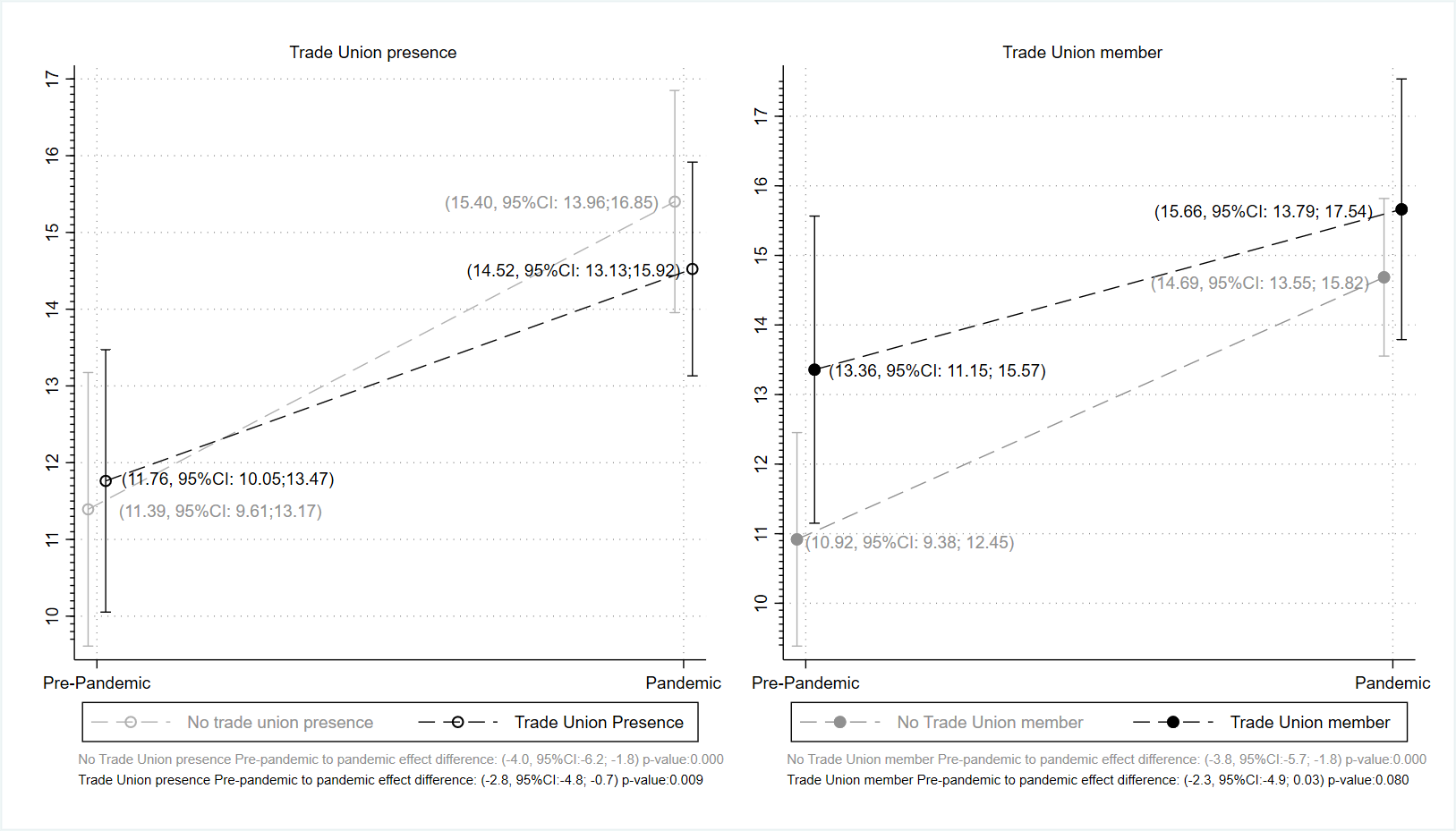


# Supplementary file S5. Trade union presence and membership and rate of change across Understanding Society (UKHLS) available waves

| **Trade union presence as percentage of the employed population** | | | | | | |
| --- | --- | --- | --- | --- | --- | --- |
|  | Percentage | | | Stability rate (t/t-1) (in percent) | | |
| Year |  | Lower 95%CI | Upper 95%CI |  | Lower 95%CI | Upper 95%CI |
| 2011 | 48.5 | 47.7 | 49.3 | - | - | - |
| 2013 | 47.8 | 46.9 | 48.6 | 81.5 | 80.8 | 82.1 |
| 2015 | 49.3 | 48.4 | 50.2 | 82.5 | 81.8 | 83.2 |
| 2017 | 46.8 | 45.8 | 47.7 | 82.7 | 81.9 | 83.4 |
| 2019 | 46.1 | 45.1 | 47.1 | 82.8 | 82.0 | 83.6 |
| 2021 | 45.8 | 44.7 | 46.8 | 83.1 | 82.1 | 83.8 |
|  |  |  |  |  |  |  |
| **Trade union membership as percentage of the employed population reporting workplace presence** | | | | | | |
|  | Percentage | | | Stability rate (t/t-1) (in percent) | | |
| Year |  | Lower 95%CI | Upper 95%CI |  | Lower 95%CI | Upper 95%CI |
| 2011 | 57.6 | 56.6 | 58.7 | - | - | - |
| 2013 | 58.6 | 57.0 | 59.3 | 79.3 | 78.4 | 80.3 |
| 2015 | 57.8 | 56.5 | 59.1 | 81.6 | 80.6 | 82.6 |
| 2017 | 56.4 | 55.1 | 57.7 | 81.1 | 80.1 | 82.1 |
| 2019 | 55.5 | 54.1 | 56.9 | 82.3 | 81.3 | 83.4 |
| 2021 | 56.5 | 55.0 | 58.0 | 82.9 | 81.7 | 84.0 |

Note: Data are weighted using cross-sectional UKHLS-provided weights

# Supplementary file S6. STROBE checklist

|  | Item No. | Recommendation | Page  No. | Relevant text from manuscript |
| --- | --- | --- | --- | --- |
| **Title and abstract** | 1 | (*a*) Indicate the study’s design with a commonly used term in the title or the abstract |  | NA |
|  |  | (*b*) Provide in the abstract an informative and balanced summary of what was done and what was found | 2 | . |
| Introduction | | | |  |
| Background/rationale | 2 | Explain the scientific background and rationale for the investigation being reported | 4 to 8 | (Introduction) |
| Objectives | 3 | State specific objectives, including any prespecified hypotheses | 8 |  |
| Methods | | | |  |
| Study design | 4 | Present key elements of study design early in the paper | 8 |  |
| Setting | 5 | Describe the setting, locations, and relevant dates, including periods of recruitment, exposure, follow-up, and data collection | 8 to 13 | Data and methods section |
| Participants | 6 | (*a*) *Cohort study*—Give the eligibility criteria, and the sources and methods of selection of participants. Describe methods of follow-up  *Case-control study*—Give the eligibility criteria, and the sources and methods of case ascertainment and control selection. Give the rationale for the choice of cases and controls  *Cross-sectional study*—Give the eligibility criteria, and the sources and methods of selection of participants | 8 to 9 and supp. File 1. |  |
|  |  | (*b*) *Cohort study*—For matched studies, give matching criteria and number of exposed and unexposed  *Case-control study*—For matched studies, give matching criteria and the number of controls per case | NA |  |
| Variables | 7 | Clearly define all outcomes, exposures, predictors, potential confounders, and effect modifiers. Give diagnostic criteria, if applicable | 8, 9, 10 |  |
| Data sources/ measurement | 8* | For each variable of interest, give sources of data and details of methods of assessment (measurement). Describe comparability of assessment methods if there is more than one group | 8, 9, 10 |  |
| Bias | 9 | Describe any efforts to address potential sources of bias | 12 | Sensitivity analyses |
| Study size | 10 | Explain how the study size was arrived at 49,915 observations | 8-9 and supp file 1. |  |

| Quantitative variables | 11 | Explain how quantitative variables were handled in the analyses. If applicable, describe which groupings were chosen and why | 9,10,11 |  |
| --- | --- | --- | --- | --- |
| Statistical methods | 12 | (*a*) Describe all statistical methods, including those used to control for confounding | 11-12 |  |
|  |  | (*b*) Describe any methods used to examine subgroups and interactions | 11-12 |  |
|  |  | (*c*) Explain how missing data were addressed | 11-12 |  |
|  |  | (*d*) *Cohort study*—If applicable, explain how loss to follow-up was addressed  *Case-control study*—If applicable, explain how matching of cases and controls was addressed  *Cross-sectional study*—If applicable, describe analytical methods taking account of sampling strategy | 11-12 |  |
|  |  | (*e*) Describe any sensitivity analyses | 12 |  |
| Results | | | | |
| Participants | 13* | (a) Report numbers of individuals at each stage of study—eg numbers potentially eligible, examined for eligibility, confirmed eligible, included in the study, completing follow-up, and analysed | Supp file 1 |  |
|  |  | (b) Give reasons for non-participation at each stage | NA |  |
|  |  | (c) Consider use of a flow diagram | Supp file 1 |  |
| Descriptive data | 14* | (a) Give characteristics of study participants (eg demographic, clinical, social) and information on exposures and potential confounders | 12-13 and supp file 3 |  |
|  |  | (b) Indicate number of participants with missing data for each variable of interest |  |  |
|  |  | (c) *Cohort study*—Summarise follow-up time (eg, average and total amount) |  |  |
| Outcome data | 15* | *Cohort study*—Report numbers of outcome events or summary measures over time | Supp file 1 and 3 |  |
|  |  | *Case-control study—*Report numbers in each exposure category, or summary measures of exposure |  |  |
|  |  | *Cross-sectional study—*Report numbers of outcome events or summary measures |  |  |
| Main results | 16 | (*a*) Give unadjusted estimates and, if applicable, confounder-adjusted estimates and their precision (eg, 95% confidence interval). Make clear which confounders were adjusted for and why they were included |  |  |
|  |  | (*b*) Report category boundaries when continuous variables were categorized | 14-16 | AME with 95%CI |
|  |  | (*c*) If relevant, consider translating estimates of relative risk into absolute risk for a meaningful time period |  |  |

Continued on next page

| Other analyses | 17 | Report other analyses done—eg analyses of subgroups and interactions, and sensitivity analyses | 16 |  |
| --- | --- | --- | --- | --- |
| Discussion | | | | |
| Key results | 18 | Summarise key results with reference to study objectives | 16 |  |
| Limitations | 19 | Discuss limitations of the study, taking into account sources of potential bias or imprecision. Discuss both direction and magnitude of any potential bias | 17-18 |  |
| Interpretation | 20 | Give a cautious overall interpretation of results considering objectives, limitations, multiplicity of analyses, results from similar studies, and other relevant evidence | 18-19 |  |
| Generalisability | 21 | Discuss the generalisability (external validity) of the study results | 17 and 19 |  |
| Other information | |  | | |
| Funding | 22 | Give the source of funding and the role of the funders for the present study and, if applicable, for the original study on which the present article is based | Done |  |

*Give information separately for cases and controls in case-control studies and, if applicable, for exposed and unexposed groups in cohort and cross-sectional studies.
